# Expansion of tandem repeats in sea anemone *Nematostella vectensis *proteome: A source for gene novelty?

**DOI:** 10.1186/1471-2164-10-593

**Published:** 2009-12-10

**Authors:** Guy Naamati, Menachem Fromer, Michal Linial

**Affiliations:** 1 School of Computer Science and Engineering, The Hebrew University of Jerusalem, Jerusalem, 91904, Israel; 2 Department of Biological Chemistry, Institute of Life Sciences, The Sudarsky Center for Computational biology, The Hebrew University of Jerusalem, Jerusalem, 91904, Israel

## Abstract

**Background:**

The complete proteome of the starlet sea anemone, *Nematostella vectensis*, provides insights into gene invention dating back to the Cnidarian-Bilaterian ancestor. With the addition of the complete proteomes of *Hydra magnipapillata *and *Monosiga brevicollis*, the investigation of proteins having unique features in early metazoan life has become practical. We focused on the properties and the evolutionary trends of tandem repeat (TR) sequences in Cnidaria proteomes.

**Results:**

We found that 11-16% of *N. vectensis *proteins contain tandem repeats. Most TRs cover 150 amino acid segments that are comprised of basic units of 5-20 amino acids. In total, the *N. Vectensis *proteome has about 3300 unique TR-units, but only a small fraction of them are shared with *H. magnipapillata, M. brevicollis*, or mammalian proteomes. The overall abundance of these TRs stands out relative to that of 14 proteomes representing the diversity among eukaryotes and within the metazoan world. TR-units are characterized by a unique composition of amino acids, with cysteine and histidine being over-represented. Structurally, most TR-segments are associated with coiled and disordered regions. Interestingly, 80% of the TR-segments can be read in more than one open reading frame. For over 100 of them, translation of the alternative frames would result in long proteins. Most domain families that are characterized as repeats in eukaryotes are found in the TR-proteomes from Nematostella and Hydra.

**Conclusions:**

While most TR-proteins have originated from prediction tools and are still awaiting experimental validations, supportive evidence exists for hundreds of TR-units in Nematostella. The existence of TR-proteins in early metazoan life may have served as a robust mode for novel genes with previously overlooked structural and functional characteristics.

## Background

Repeated domains in proteins are associated with a broad spectrum of functions. They have originated from repetitive DNA elements, including transposons, retroviruses, and other parasitic elements that impact genome dynamics. While most of these events are deleterious, some may lead to new proteins or protein domains, as was shown for Alu repeats in primates [1]. Repeated sequences may account for the majority of the genetic material in many eukaryotic genomes, among them the human genome [2]. Some repeated sequences may result in open reading frames (ORFs) of substantial length. If these sequences are integrated into an actively transcribed region, subsequent events such as recombination and gene conversion can spread this repeated sequence, resulting in fast amplification of the original ORF [3].

Tandem repeats (TR) in proteins are of various types. At one extreme are those based on a single amino acid repeat. Slippage during DNA replication could yield a repeated unit of exactly 3 nucleotides, resulting in the gain of a single amino acid. It has also been found that most mono-amino acid repeats in primates are encoded by GC-rich isochores [4], but there exists a strong purifying selection against such short repeats at the DNA level [5]. The over-represented mono-amino acid repeats in eukaryotes are glutamine (Q), proline (P), glutamic acid (E), asparagine (N), aspartic acid (D), and serine (S). Mono-amino acid repeats appear mostly in transcription factors and protein kinases [6,7], where they occupy flexible regions and loops. Interestingly, the number of repeated glutamines (Q) in key proteins correlates with the severity of Huntington disease and Fragile X Syndrome [8].

A TR protein may contain short repeat units (3-4 amino acids), which occur tens and hundreds of times in tandem. In such cases, the TR units typically form an extended structure, with this phenomenon quite common in structural proteins such as collagens, keratins, antifreeze proteins, and spider silks. Such structural proteins and others, like the FG-rich proteins of the nuclear pore [9], possess unique biophysical and thermodynamic properties.

About 250 InterPro entries [10] are characterized as *'repeats'*. These are protein regions that do not fold into a globular domain on their own [11]. Often, several of these repeats are needed to form a globular domain. Known examples for repeated domains that provide the scaffold of the protein are ankyrin (33 amino acids), keltch (50 amino acids), TRP (34 amino acids) and armadillo (40 amino acids) repeats. The stable 3D structures of these domain families rely on the rearrangement of their repeated units; for example, the repeat unit of WD40 represents a four-stranded β-sheet arrangement. In fact, many of the repeated domains are rich in β-sheets, where these proteins typically participate in a network of interactions such as in cell-cell contact, pathogen adhesion [12], and cell signaling.

Several methods and tools for detecting repeats in large protein databases are available [13-16]. TRs that are characterized by a predefined periodicity are categorized in the TRIPS database [14]. Tools such as HHRep [17] and PEPPER [18] efficiently address genome-scale search for multiple repeats in proteins. The latter is suitable for analyzing sequences of fibrous proteins. TRPpred is a tool that was optimized for identifying repeated units in several protein families including TRP [19]. The Xstream server [20] is a sensitive tool for detecting TRs on a large-scale. Furthermore, it permits flexibility in defining TRs, by fine-tuning the parameters to successfully detect TRs in DNA or protein sequences. The Xstream server does not tolerate non-conserved spacers between the TR units. Therefore, proteins having multiple repeated domains (e.g., WD40, Kelch) do not necessarily fulfill the Xstream definition of TR-proteins.

Currently, most multicellular model organisms that have been studied come from Bilateria. An important step toward the study of metazoan evolution comes from the eumetazoan phylum Cnidaria, a lineage that includes corals, sea anemones, jellyfish, and hydroids. A genomic perspective on the metazoan origin comes from the recently sequenced genomes of the choanoflagellate *Monosiga brevicollis *[21], the starlet sea anemone *Nematostella vectensis*, and *Hydra magnipapillata *[22]. The addition of genomic information from several Porifera (sponges) contributed to the reconstruction of the evolutionary position of Metazoa with respect to Fungi [23]. Surprisingly, many genes that were initially regarded as vertebrate-specific have in fact been identified in the *N. vectensis *genome [22]. These genomes allowed researchers to estimate the branch linking Cnidaria and the Bilateria to have occurred 650-750 million years ago [24].

Herein, we focus on the prevalence of TRs in the sea anemone proteome. When comparing it to other genomes, a surprising enrichment in the size and complexity of its TRs is evident. The diversity of the TR sequences from *N. vectensis *is considerably higher than that identified in any other multicellular eukaryotic genome. The properties of the TRs in *N. vectensis *are discussed in view of their evolutionary potential as a source for gene novelty. We discuss the advantages of expansion and increased diversity of TR-proteins as a robust mode for constructing new genes with unique functional properties.

## Results

### Scarlet sea anemone proteome is exceptionally rich in tandem repeats

**Figure 1:**
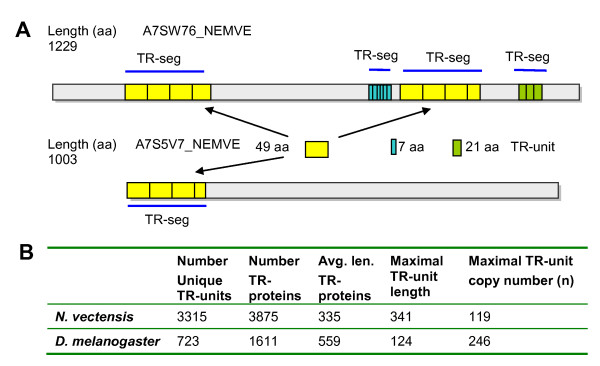
**TR-containing proteins from *N. vectensis***. Graphical representation of two TR-containing proteins: A7SW76 and A7S5V7 (UniProt). Proteins contain TR with a unit length of 49 amino acids (yellow, TR-unit). This TR-unit appears twice on A7SW76, while the other two TR-units are unique to A7SW76. These TR-units are of length 7 and 21 amino acids (blue and green), respectively. For consistency, we count the number of unique TRs in a genome (i.e. unique non-overlapping sequences that fulfill the TR definition). TR-proteins sum all proteins that contain at least one TR-segment (TR-segment, TR-seg). The minimal TR-segment is composed of at least 3 successive TR-units. (B) Statistical comparison of *N. vectensis *and *D. melanogaster *TR-proteomes.

An increased number of sequenced genomes led to a revised view of the origin of metazoan life. The eumetazoan clade contains taxa with nervous systems and muscle cells: Cnidaria, Ctenophora, and Bilateria [25]. In addition, the complete proteome of the starlet sea anemone, *Nematostella vectensis*, provides insight into gene invention dating hundreds of millions of years ago back to the last common Cnidarian-Bilaterian ancestor [26]. The *N. vectensis *genome allows us to examine the evolution of an ancestral genome that is rich in TRs. Examples of TR-proteins that fulfill this definition are shown in Figure 1A. Proteins A7SW76 and A7S5V7 (UniProt IDs) share a repeated unit of 49 amino acids (yellow box) with a copy number of 3.5 and 4. In addition, two other repeated units are found in A7SW76, with a copy number of 6 (7 amino acids unit) and 3 (21 amino acids unit). The remaining portions of the proteins are not identified as TRs. TR-units are defined by at least 3 repeated units, each with a minimal length of 3 amino acids, with no intervening sequences. These are non-overlapping sequences that are identified according to the Xstream tool [20] (see Methods). The number of unique TRs may differ from that of TR-proteins as the same TR-unit might occur in numerous proteins and the same TR-unit often appears in several segments on a particular protein (Figure 1).

We compared the occurrence of protein TR sequences in *N. vectensis *in view of other complete proteomes, including fly (*D. melanogaster*), human, mouse, frog, and more. We restricted our analysis to those TRs for which all repeats share >80% sequence identity with the respective consensus repeat sequence (see Methods).

The complete proteome of *N. vectensis *includes 24,906 predicted proteins, where half of these are marked as fragments. The number of proteins identified with TRs was as high as 3875, of which 3315 are unique TR-units. When comparing the TRs in *N. vectensis *and other proteomes, the most striking difference is in the number of unique TRs. In *N. vectensis*, ~16% of all protein sequences are TR-containing proteins. When employing the same parameters, TR containing sequences account for only 3% of the *Drosophila melanogaster *proteome (Figure 1B).

An exhaustive comparison of the TR properties for representative proteomes, spanning an evolutionary range from *N. vectensis *to human, was performed. The selected organisms represent major evolutionary branches, covering worm (*C. elegans*), insects (fly, bee and beetle), plant (Arabidopsis), vertebrate (chicken, frog), marine chordata (Ciona), cnidaria (Hydra), mammals (human, mouse), metazoan parasite (Leishmania) and choanoflagellate *(M. brevicollis)*.

**Figure 2:**
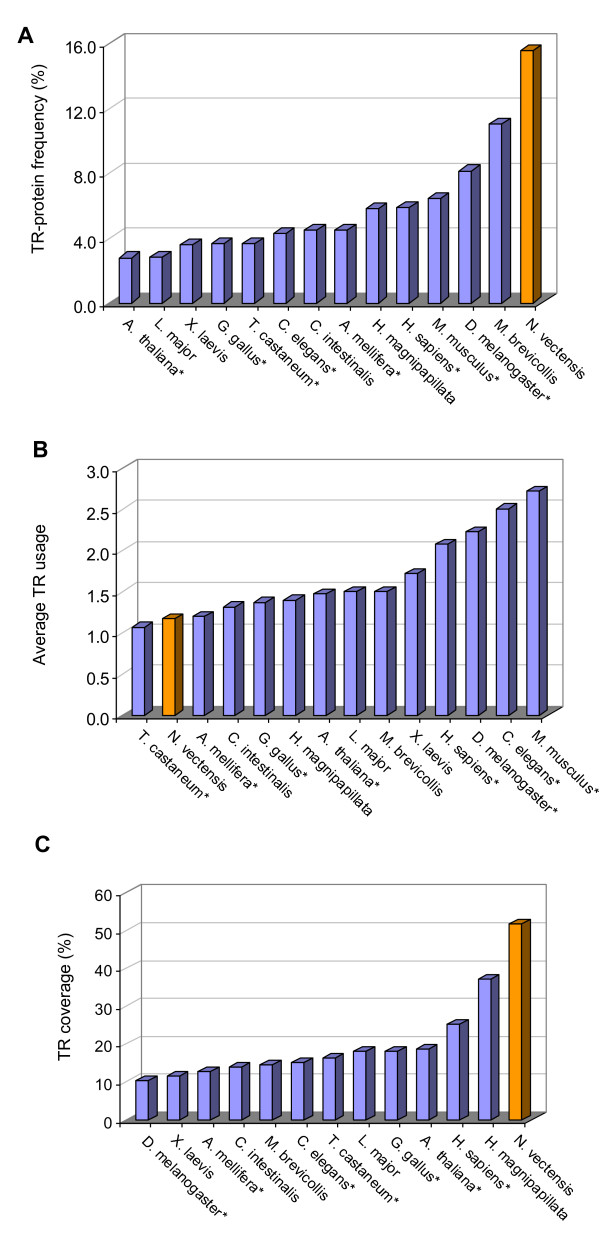
***N. vectensis *TRs relative to representative proteomes along the metazoan evolutionary tree**. (A) The fraction of TR-proteins within the proteome tested for 14 model organisms. Representative organisms include plant, insects, worm, sea squirt, frog, and more. The relative fraction of TR-proteins in *N. vectensis *is nearly double that of fly and almost 5 fold higher than the plant representative. (B) The usage of the unique TRs in the proteins. In mouse, worm, fly, and human, on average, each TR is used in >2 distinct proteins. However, for *N. vectensis*, this ratio is close to one. (C) The length of the TR-segment relative to the length of the protein that contains it (denoted as coverage). The coverage is < 20% in most organisms excluding *N. vectensis*.

When the fraction of TR containing proteins (TR-proteins) relative to the total Nematostella proteome is considered (Figure 2A), we noted a 2-5 fold enrichment in the fraction of such proteins relative to the other organisms tested.

### Most *N. vectensis *tandem repeat units are unique

While the fraction of TR-proteins in *N. vectensis *is higher than in any other tested organism, the usage of any particular TR is typically restricted. Most TR-units are used only once in *N. vectensis *(Figure 2B). In mouse and human, each TR appears on average in 2.7 and 2.1 proteins, respectively. For example, in the mouse proteome, there are ~3000 TR-proteins, yet they are composed of only ~1100 unique TRs. While the genomes of human and mouse are quite active in reusing their repeated sequences [27], no clear view was presented for the dynamics of TRs in *N. vectensis *[22]. Moreover, the fraction occupied by a TR within the TR-protein (i.e., TR-coverage) is substantially higher in *N. vectensis *(Figure 2C). This extreme TR-coverage (50%) reflects the fact that almost half of *N. vectensis *proteins are marked as fragments due to missing exons (and failures of genome prediction tools). The 25% coverage measured for human (Figure 2C) and mouse (not shown) is still higher than the coverage determined for the other organisms tested, mainly insects and frog.

### Characterizing the properties of TR-containing proteins from *N. vectensis*

**Figure 3:**
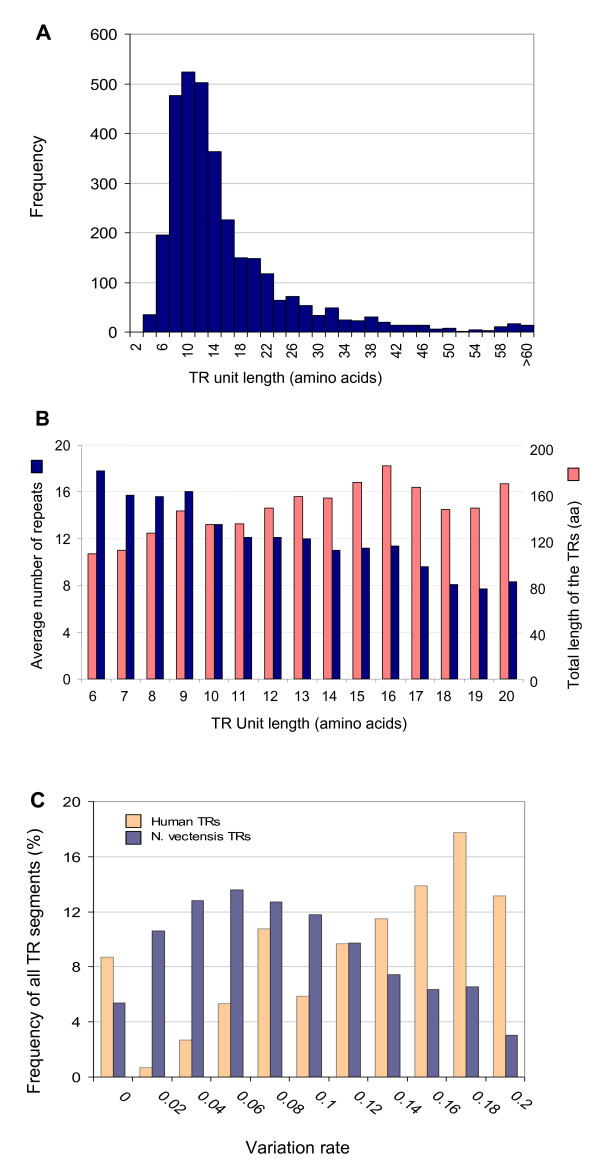
**Properties of the TRs in *N. vectensis***. (A) The distribution of the TR-unit length for all 3212 unique TR sequences from *N. vectensis*. A unit length of 10-12 amino acids is most frequent. The tail of the length distribution (of repeats longer than >60 amino acids) is not shown. (B) The relationship between TR-unit length, total length of the TR-segment (in pink) and the average number of repeats (copy number, blue). As the length of the TR-unit increases, the copy number of the repeats decreases. (C) Comparison of the variation rate within TR-segments from *N. vectensis *(blue) and human (pink). Variation rate is measured up to a 20% cumulative difference in the sequence of the TR-segment relative to the consensus TR-unit. For details, see Additional file 1.

The >3300 TRs of *N. vectensis *were statistically analyzed to determine the most prevalent repeat properties in terms of number of TR units, their length, and the overall regions they occupy. Figure 3 shows the abundance of these TR-units. It is evident that a unit length of 10-12 amino acids is most prevalent (Figure 3A). Furthermore, an inverse correlation is observed between the number of TR-units within a TR-segment (defined in Figure 1) and the length of that basic unit (tested for a range of 6-20 amino acids). Thus, in most cases, the total region that is occupied by TRs in *N. vectensis *remains within the range of 120 to 180 amino acids (Figure 3B, pink). The average length of a TR-segment in a protein is 153 amino acids. Figure 3C shows the accumulated level of variations relative to the TR consensus, where TRs with deviations ranging from 0 to accumulated changes in 20% of its amino acids (marked 0.2) are analyzed. The TRs from human and *N. vectensis *demonstrate clear differences. While most TRs in human have diverged substantially, this is not the case for *N. vectensis *(peaks at 0.06). It is possible that TRs in human and *N. vectensis *differ in their tendency to accumulate and maintain variations. A substantial number of TRs in human (229) and in *N. vectensis *(235) shows no variations (marked 0) but most of them (80%) are short TRs (<6 amino acids). An attractive idea suggests that the higher variation observed in mouse (not shown) and human (Figure 3C) coincide with the high usability of each TR-unit in human and mouse but not in *Nematostella *(Figure 2B). A similar trend was proposed for the accelerated evolution of duplicated paralogous genes [28]. A similar analysis performed for the ~1200 TR-segments of Hydra indicated an intermediate rate of variation (Additional file 1).

We expanded the comparative study to proteomes that are evolutionarily closer to *N. vectensis*, such as Hydra. Recently, a Hydra genome (*H. magnipapillata*) was sequenced and a predicted protein set of 17,586 models was presented (Craig Venter Institute, ~6X sequence coverage, 1.3 Gb genome). The fraction of unique TR-units within the Hydra complete proteome is ~5% (Table 1).

**Table 1:** TR properties in 14 representative proteomes

	Number ORFs	Number unique TRs	Number TR-proteins	Avg. len. TR-protein	% fragments^a^	Maximal copy number (n)	Maximal TR length
C. intestinalis***	1,496	51	67	480	32.3	30	44
L. major***	8,317	158	237	631	1.5	1385	771
X. laevis***	15,772	329	565	429	17.9	55	84
T. castaneum*	9,766	334	357	528	29.5	96	189
A. mellifera*	9,257	348	417	510	42.5	55	105
G. gallus*	18,529	493	674	471	29.4	56	366
A. thaliana*	31,921	602	888	409	11.8	46	120
C. elegans*	38,096	655	1641	464	5.7	230	903
M. brevicollis***	9,269	690	1017	597	10.5	141	135
D. melanogaster*	19,789	723	1611	559	17.6	246	124
H. magnipapillata**	17,586	882	1016	422	N.A.	97	472
H. sapiens*	34,180	967	2008	481	26.8	238	1201
M. musculus*	46,892	1106	3007	478	23.3	131	515
N. vectensis***	24,906	3315	3875	335	46.8	119	341

^a^The fraction of fragments is based on the UniProtKB collection.

* Complete proteomes as extracted from the Xstream resource; ** Data from NCBI based on Craig Venter Institute genome project. *** Data from UniProtKB

The difference in the absolute number of proteins and the fraction of proteins marked as 'fragments' (Table 1) is a proxy for the knowledge and annotation quality (exceptions are missing annotations for *L. major *and Hydra). The high fraction of partial proteins (i.e., 47% 'fragments' for *N. vectensis*) is consistent with their shorter average length (Table 1). We included in the analysis *Leishmania major*, a protozoan parasite that underwent rapid evolution and for which many proteins are known to contain TRs. Table 1 shows that the exceptionally high proportion of proteins with TRs in *N. vectensis *is a unique property of this organism and has not been reported to such an extent even in protozoan parasites.

To ensure that the trends seen in Table 1 do not solely reflect the poor quality of the assembly and protein annotations reported [29], we repeated the analysis but eliminated all sequences that include undefined nucleotides (indicated by nucleotide 'x') or sequences that could not match exactly their transcripts (see Methods). Even after such filtration, the fraction of TRs for *N. vectensis *still remains exceptionally high (11% of the entire proteome).

### Amino acid composition of *N. vectensis *tandem repeats

**Figure 4:**
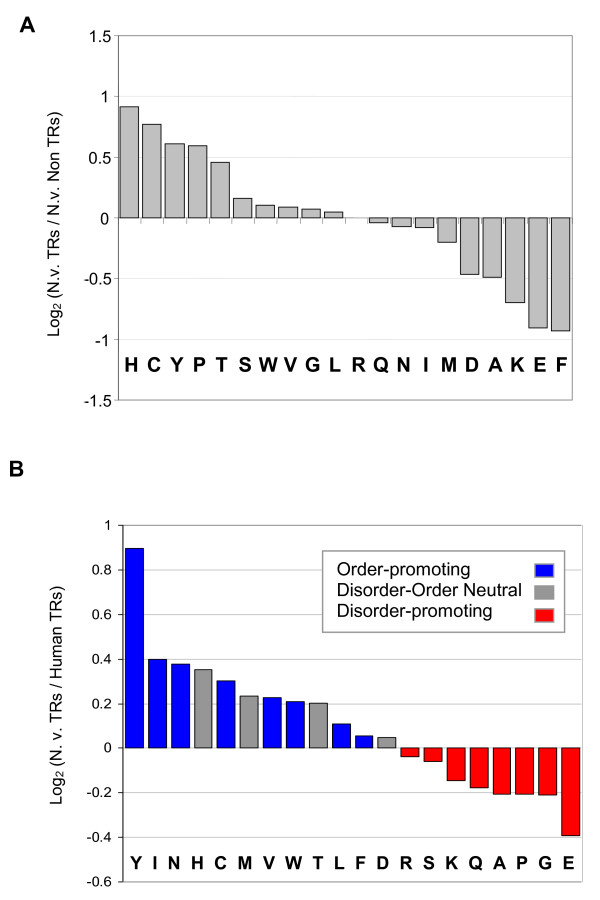
**Amino acid composition in TR-segments**. (A) Composition of amino acids in TR-segments relative to non-TR proteins from *N. vectensis*. The over-represented and under-represented amino acids are shown. (B) The TR-proteins from *N. vectensis *were compared to human TR-proteins. Amino acids were colored according to the partition of the disorder propensity [52]. Disorder-promoting residues (A, R, S, Q, E, G, K, P) are colored red, order-promoting residues (N, C, I, L, F, W, Y, V) are colored blue, and disorder-order neutral residues (D, H, M, T) are colored gray.

We analyzed the over-representation and under-representation of amino acids in TR-proteins, in comparison with all other *N. vectensis *proteins. We found that several amino acids, most notably histidine (H), cysteine (C), tyrosine (Y), proline (P), and threonine (T) were enriched, with C and H being the most significantly so (Figure 4). On the other hand, we observed that polar and charged amino acids are strongly depleted in the TR sequences. Specifically, the most depleted amino acids are phenylalanine (F), glutamic acid (E), and lysine (K). Similar enrichment and depletion in amino acid preference were evident when compared to the SwissProt database (~400,000 sequences). In addition to the distinct biophysical properties of these under-represented amino acids, it is of interest that their codons tends to be AT rich and they are mostly coded by a limited number of codons (2 codons each).

A significant difference in amino acid composition exists between *N. vectensis *TR and non-TR proteins (Figure 4A). We thus tested whether the amino acid composition in TRs of *N. vectensis *is similar to that of other organisms. Specifically, we compared the *N. vectensis *TRs to the ~970 unique TRs from human. When comparing the TR sequences (Figure 4B), a relative enrichment in tyrosine (Y) is evident, and to a lesser extent C, I, L, and V. Interestingly, relative to the human repeats, the TRs from *N. vectensis *are more enriched with amino acids that tend to form ordered structures (Figure 4B, blue), suggesting that these repeats may be better suited to form structural units. Similar trends were evident in comparisons with other vertebrate TR-proteins. The most dominant predicted secondary structure associated with the TR repeats is the β-sheet (not shown).

### Sequence robustness of tandem repeats revealed by multiple open reading frames

Among the 24,906 ORFs predicted for *N. vectensis *[22], 3875 TR-proteins were selected (Table 1). We tested the extent of valid alternative reading frames among this large set of TR-proteins. In fact, due to the shortage in experimental evidence (ESTs and cDNAs), and the absence of any direct protein information, the reading frame may not be correct. Typically, computational inference for a specific frame is based on an appropriate Kozak sequence near the initiating methionine, on the knowledge of codon usage bias, and on conservation criteria from homology and paralogy. At present, ~50% of the TR-proteins lack an initiating methionine and a similar fraction (45%) holds for the rest of the predicted proteins.

**Figure 5:**
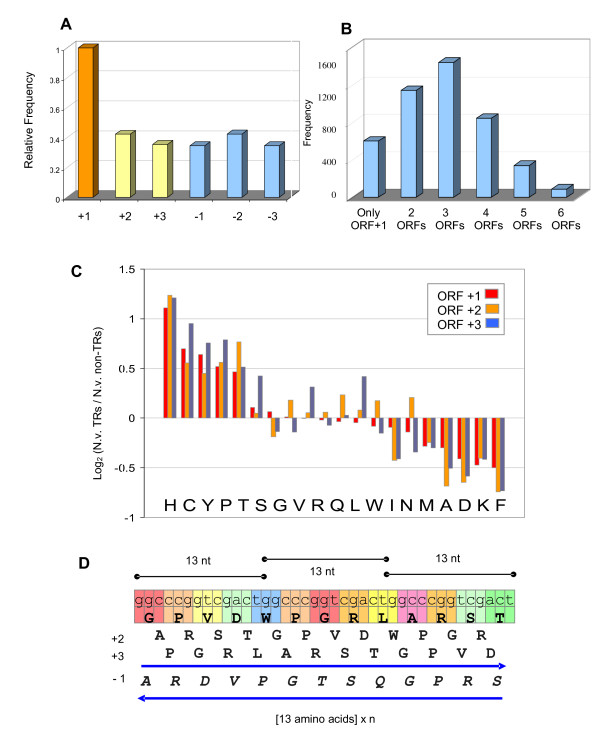
**Multiple valid ARFs in TR-segments**. (A) Frequency of valid ARFs (i.e., an ORF without stop codons) for all 6 possible reading frames for 4437 TR-repeated segments ORFs from JGI *N. vectensis *proteome. The average frequency of valid ARFs in any of the alternative frames is 0.375. The frequencies of all reading frames were normalized so that the original frame (ORF +1, 100%). (B) For each of the 4437 TR-proteins, number of valid ORFs (including the original frame). For example, there are 800 TR-proteins with 4 valid ORFs (including ORF +1). (C) Over-represented and under-represented amino acids for all three ORFs (ORF +1 indicates the original ORF). (D) A scheme for a repeat segment in which each reading frame translates to a nearly identical protein (differing only at the beginning and end of the sequence). In this example, the reverse complement frames are also valid (i.e., do not encounter a stop codon) throughout the sequence. The arrows indicate the directionality of the transcript.

We analyzed the properties of the alternative reading frames for TR-proteins. For this analysis, the set of all TR-segments from *N. Vectensis *was compiled (4437 segments, JGI proteome). For each such TR-segment, we inspected all alternative reading frames (ARFs), a total of 22,185 potential ORFs. Averaging over all 5 frame shifts, 37.5% of these ARFs do not contain a stop codon (Figure 5A), i.e., they adhere to the minimal definition of being valid ORFs. To put this result within a statistical context, we performed a simulation for 4437 random ORF sequences, generated based on the nucleotide composition of the coding sequences of the *N. vectensis *TR-proteins. In this random simulation, only 4.3% of the ARFs did not contain a stop codon, thus hinting at the possible significance of this beneficial use of repeated sequences to yield valid ARFs. Somewhat surprisingly, the high fraction of valid ARFs also carried over for those of the reverse complement strand (Figure 5A). In our analysis, we found that only ~600 TR-proteins (14%) can be read exclusively in the annotated reading frame. On the other hand, for most of the sequences, there are at least 2-3 additional ORFs (Figure 5B), many of which are potentially long. For example, when limiting the analysis to ARFs (ORF +2 or ORF +3) with length >500 nucleotides, 107 such instances were found (Additional file 2).

An analysis of the TR-proteins showed that the overall amino acid composition is rather similar for all 3 reading frames on the coding strand, when the alternative frames are open; (Figure 5C, p-value for paired t-test <0.007). Note that in cases when the reverse complement frames are also valid, the translated sequences are also often similar in composition to the original frame (see Discussion). It is important to note that there is a unique scenario, for which repeats of a nucleotide unit will necessarily result in the same amino acid sequence for all 3 reading frames on a strand, with cyclic shifts only in the start and end of the sequence. Figure 5D demonstrates an example where a repeat of 13 nucleotides leads to a TR-unit of 13 amino acids. This phenomenon is actually a byproduct of the basic repeat unit (at the DNA level) having any length that is not a multiple of 3. We found that only for 18% of all TR-proteins (806 instances) was this phenomenon present. Clearly, in such cases, the amino acid composition will remain fixed. Nonetheless, we found the composition to be fixed for almost all TR-proteins (Figure 5C).

### Limited conservation of *N. vectensis *TR-units along the phylogenetic tree

**Figure 6:**
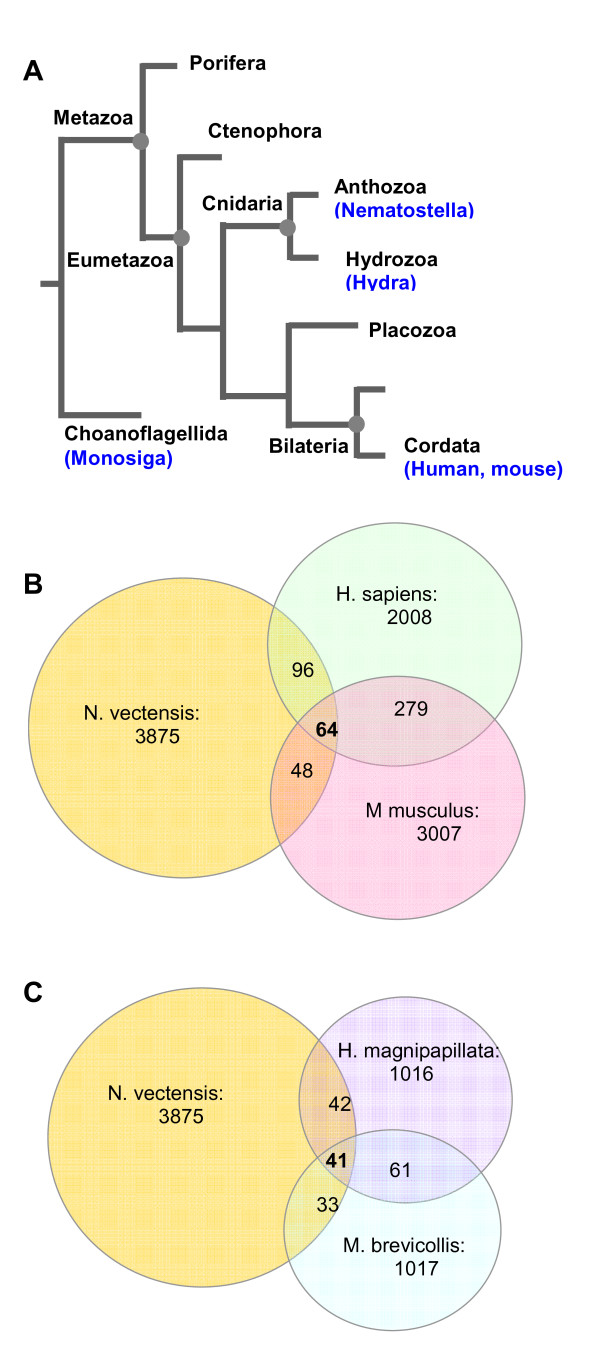
**Evolutionary conserved *N. vectensis *TR-units**. (A) A schematic phylogenetic tree showing the main branches in metazoan origin. Proteomes that are compared are indicated in blue. (B) *N. vectensis *shares 160 TR-units with human and 112 TR-units with mouse. Mouse and human share 343 TRs, among which 64 are shared by all three organisms. (C) *Hydra magnipapillata *TR-proteins were compared to *N. vectensis*. 83 TR-units are shared between these two organisms. *N. vectensis and M. brevicollis *share 74 TR-proteins. 41 proteins are shared among all three organisms. For the list of shared proteins, see Additional file 3.

We tested to what extent the 3300 TRs from *N. vectensis *is evolutionarily conserved. We focused on representative proteomes for comparison with *N. vectensis*. Figure 6A shows a tree view of the major branches from the metazoan-fungi separation and within the metazoa kingdom. The Venn diagrams of TR-proteins indicate the number of TR-proteins that are shared among human, mouse, and Nematostella (Figure 6B). A broader evolutionary perspective is presented by comparing Nematostella with Hydra (Cnidaria) and Monosiga (Figure 6C). Shared TR-proteins are defined by the identity of their TR-units, as calculated by Xstream [20]. We noted that even human and mouse share only 343 TR-proteins, while the overlap between *N. vectensis *and human and *N. vectensis *and mouse is even lower (160 and 112, respectively). The analysis of unique TRs shows that 6% of the unique TRs from *N. vectensis *are also found in human and mouse (Figure 6B). Among these, only 64 TR-proteins are shared among all 3 proteomes. While some of the TR-units that are shared among the tested proteomes are rather long, ~50-60% of them are shorter than 7 amino acids. A similar comparison relative to the Hydra proteome indicated that 10% and 7.4% of the TR-proteins are shared with Hydra and Monosiga, respectively (Figure 6C). Only 20 TR-proteins are conserved among all 5 tested species. We concluded that a limited number of TR-units are shared in evolution and the expansion of the TR-proteome is indicative of Nematostella and to a much lesser degree of Hydra. For more details, see Additional file 3.

### Expression of TR-proteins from *N. vectensis*: the multi-ubiquitin proteins

Table 2 summarizes instances of TRs that are shared among *N. vectensis*, human, and mouse and additional organisms. Among the longest TRs that were identified in human, mouse, and *N. vectensis *(with almost no variability in their sequence) is a repeat of 76 amino acids that represents multi-ubiquitin proteins. For example, *D. melanogaster *has 4 proteins with such TR-units, with copy number (n) of 4 (Q8MT02), 7 (Q9W418) 10 (A4V1F9) and 14 (Q8MSM5). In *N. vectensis*, this TR-unit is detected in 2 independent sequences with a copy number of 3 and 7 units (A7SV54 and A7SUP6, respectively). This ubiquitin domain of 76 amino acids appears within a wide spectrum of the taxonomical tree. Representative proteins containing this TR, with n = 3 to 17, are shown in Table 2. Hs1-Cortactin is a TR of 37 amino acids that appears with n = 3 to 7. The 114 amino acid repeat of Calx-beta appears in 7 protein sequences in *N. vectensis*. It is widely spread throughout the taxa, ranging in copy number from 3 to 41. The Calx-beta motif is present in the cytoplasmic domains of Na-Ca exchangers and in integrin-β4, which mediates signaling across the plasma membrane (see Additional file 4). In all these examples, the TR units are those reported by the Pfam family collection [30]. All *N. vectensis *examples listed in Table 2 are supported experimentally and confirmed by EST expressions from embryo, larva, and unfertilized egg (not shown).

**Table 2:** Representatives of TRs shared between *N. vectensis *and other organisms.

	TR copy number (n)	UniProt ID	Length	# of seq
**Polyubiquitin (TR-unit 76 aa) **				
C. albicans*	3	Q5ADS0_CANAL	229	91
*A. thaliana*	4	Q7GAQ5_ARATH	324	78
*S. paradoxus*	5	A2RVC2_SACPA	389	73
*S. japonicum*	6	Q5DGA0_SCHJA	457	22
***N. vectensis ***	**7 **	**A7SUP6_ NEMVE**	**533**	**21**
*A. thaliana*	8	Q39256_ARATH	631	10
*T. brucei*	9	Q383T7_9TRYP	685	5
*C. briggsae*	10	Q61L84_CAEBR	762	6
*L. major*	11	Q4Q165_LEIMA	837	2
*B. mori*	12	Q9XXZ6_BOMMO	913	1
*L. braziliensis*	13	A4HPM0_LEIBR	992	2
*D. melanogaster*	14	Q8MSM5_DROME	1067	2
*H. sapiens*	17	Q59EM9_HUMAN	1309	1
**Hs1-Cortactin (TR-unit 37 aa)**				
*S. japonicum*	3	Q5C1Z2_SCHJA	177	1
*D. melanogaster*	4	Q9VDF4_DROME	339	13
*T. nigroviridis*	5	Q4S596_TETNG	537	3
*M musculus*	6	Q8BNA5_MOUSE	509	12
***N. vectensis ***	**7 **	**A7RF47_NEMVE**	**534**	**11**
**Calx-beta (TR-unit 114 aa)**				
*T. nigroviridis*	3	Q4T3S8_TETNG	1122	6
*M. prolifera*	4	O16859_MICPR	825	7
*M. musculus*	5	FREM2_MOUSE	3160	11
***N. vectensis***	**6**	**A7SVI8_NEMVE**	**742**	**4**
*V. splendidus*	11	A3URD5_VIBSP	1599	1
*M. sp. pe36*	13	A6FHE3_9GAMM	2950	1
*M. prolifera*	15	O16857_MICPR	2205	1
***N. vectensis***	**17**	**A7SI34_NEMVE**	**2271**	**1**
*D. donghaensis*	22	A2TVC1_9FLAO	2755	1
***N. vectensis ***	**41 **	**A7SVI9_NEMVE**	**4953**	**1**

Tandem repeat proteins shared with representative *N. vectensis *proteins (in bold face). For detailed examples, see Additional file 4.

### A functional perspective of *N. vectensis *repeats

The fraction of Pfam and InterPro entries that are represented among all *N. vectensis *proteins is slightly lower than that for well-studied genomes (68% compared to 77% for all proteins in UniProt). When the set of TR-proteins is considered, only 31% appear with a Pfam entry that is repeated at least twice. Thus, most of the TR-proteins are undefined by Pfam and InterPro databases. Of course, the strict requirement of tandemness in the TRs excludes proteins with non-conserved linkers between domains (e.g., Annexin repeats) from the analysis.

**Figure 7:**
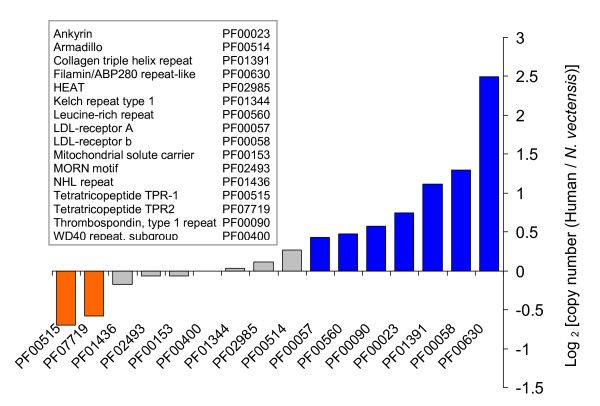
**Pfam repeated domains in *N. vectensis *proteome**. Pfam repeat domains based on *N. vectensis *InterPro annotations. Only Pfam entries with >20 proteins are listed. The histogram indicates the log-ratio of copy number for a particular TR-unit in human and *N. vectensis*. Significant differences are colored. For details and for a full list of all Pfam repeat entries in *N. vectensis*, see Additional file 5.

We thus set out to test the appearance of repeats in the *N. vectensis *proteome in view of the 252 repeat types that are reported by InterPro (Additional file 5). Most of the repeats that are supported by Pfam (63%, see Methods) are not found in *N. vectensis*. For those found in *N. vectensis*, we focused on the 16 repeat types that are represented by at least 20 proteins in *N. vectensis*. When compared to humans, no marked difference in copy number is shown for most of these repeats (Figure 7). For 2 such repeat domains, *N. vectensis *exhibits a moderate preference for a higher copy number. For almost half of the instances, the opposite tendency is detected. Since many of the *N. vectensis *proteins are incomplete (i.e., annotated as fragments), this analysis may underestimate the actual copy number of the TRs in the full sequences. We conclude that, overall, the copy numbers for the Pfam repeats that are well-represented in *N. vectensis *are rather similar to the copy numbers of these repeats in human (Figure 7).

### Evolutionary divergence rate for tandem repeat units

The large number of TR-proteins in *N. vectensis *and the observation that they are mostly uniquely used in its proteins raises the question: which evolutionary forces act on such repeats? We set out to study whether the evolution of these tandem repeats has been subject to neutral, purifying, or positive selection. We thus applied an analysis based on the ratio of asynonymous to synonymous substitutions (Ka/Ks ratio). Due to the shortage of experimental evidence for many of the predicted proteins from *N. vectensis*, we limited this analysis to the TR-proteins that are supported by ESTs (obtained from JGI genome center). For the 94 sequences analyzed, ~40% of them exhibit a ratio of Ka/Ks > 1.0, while ~50% show the opposite trend. Among the instances of TR-proteins with Ka/Ks>1, several of them seem to exhibit extremely high ratios, which strongly supports the notion of positive selection on these tandem repeats.

## Discussion

### *N. vectensis *proteome quality and missing annotations

All analyses we performed for the *N. vectensis *and *H. magnipapillata *TR-proteins are susceptible to the quality of assembly from the shotgun libraries [29]. The genome of *N. vectensis *is composed of a large number of disconnected contigs. Transposable elements constitute ~26% of the assembled genome, and they are associated with ~500 families that are considered evolutionary young. The number of DNA transposon types found in the sea anemone is the highest among eukaryotic species [31].

In *N. vectensis*, only 146,000 ESTs were used to support the proteome annotation. These ESTs led to the validation of ~8000 protein sequences. In addition, ~15,000 paired clone end sequences were generated, resulting in 27,273 predicted gene models. It has been estimated that about a third of the predicted proteins may be subjected to future annotation efforts [22]. We conducted our analysis on 24,906 proteins that are supported by UniProtKB (~91% of JGI predicted proteins). About 2000 TR-proteins are reported following an analysis using stricter criteria (for sequences that do not contain any undefined nucleotides or gaps in the TR consensus). Currently, collections from Cnidaria ESTs (sea fans and soft coral) are available: *Anemonia viridis *(~40,000), *Metridium senile *(~30,000), and *Acropora palmate *(~14,000); and *Porites astreoides*, *Aiptasia pallida*, *Acropora millepora *with ~10,000 ESTs each. We expect that the coverage of TRs in *N. vectensis *(Figure 2C) will be revised upon improved annotations and additional experimental data. We are currently testing the expression of predicted TR-proteins from *N. vectensis*.

### DNA repeats as a source of novel coding sequences

Most multicellular eukaryotic genomes contain repetitive sequences. These sequences provide the basis for the study of genome dynamics, recombination, and gene conversion [32]. In general, DNA repeats are believed to serve as a principal source of gene novelty. Repetitive nucleotide sequences such as micro- and mini-satellites have been subjected to rapid gains and losses throughout evolution [15].

A survey on internal repeats in protein sequence databases [33] indicated that eukaryotes have a 3-4 fold higher incidence of internal repeats, as compared to prokaryotes or archaea [34]. Furthermore, long proteins often resulted from the concatenation of internal TRs. We propose that the *N. vectensis *TR basic architectures are attractive for creating new proteins through recombination and copy number adjustment. For a simple sequence (devoid of TRs) above a certain length, it is rare to detect valid ARFs. Usually, only one ORF (the correct one) can be found, while frame shifts of this ORF are unavoidably punctuated by stop codons. However, we noted that for ~800 TR instances, both ARF frame +2 and frame +3 are valid as ORFs.

It was recently suggested that ~40 long overlooked ARFs are expressed in humans [35], in addition to several previously studied ARFs (GNAS1, XBP1, and INK4). When we applied similar criteria to identify ARF candidates among *N. vectensis *TR-proteins, 107 such instances were found (Additional file 2). The average fraction of valid ARFs for the TR-proteins in *N. vectensis *is disproportionately high (37.5%), when considering only the size distribution of these proteins. The analysis of all TR-proteins shows that all 3 frames provide a rather similar overall picture (p-value < 0.007, Figure 5C) of amino acid composition, even when this is not mathematically guaranteed.

The probability for a stop codon (in alternative frames and antisense strand) decreases as GC content increases [36]. In Nematostella TR-proteins, the strong bias towards multiple ORFs was not associated with high GC content. The GC content for the TR-units is similar to that of the proteome as a whole (47.4%). For humans, the GC content of the entire proteome is higher (52.7%) and similar to that of its TR units (51.9%). We conclude that the length of TRs and their complexity is not a reflection of the GC content.

### Tandem repeats with structural and functional characteristics

The energetics and kinetics of TR-containing proteins provide new insights into folding rates and protein stability [37]. In *N. vectensis*, the TR-units are of an average length of 10-12 amino acids (Figure 3A), but a TR-segment is of ~150 amino acids on average (Figure 3B). This is in the range of a foldable unit and the size of most structural global units in proteins [38]. For most TRs, there is no functional or structural information. In fact, the annotation of 'predicted protein' covers 96% of the *N. vectensis *proteins and 99.9% of the TR-proteins.

We tested the TR segments of *N. vectensis *for their secondary structure propensities using the Jpred3 server [39]. For this analysis, we used all valid ARF sequences that were at least 100 amino acids long (296 such sequences in total). Since Jpred, as other prediction methods, strongly rely on homology through PSI-BLAST, it was not surprising that for most ARFs, no globular homolog was found. On average, 83% of the positions were predicted as disordered coils (C), 7% of the positions predicted as beta sheet (E), and 10% as alpha helix (H). Nonetheless, some of these ARFs (20 of them) were predicted to have between 30% and 74% beta sheet, and others (25 ARFs) 52%-97% alpha helix. Interestingly, most InterPro-defined repeat domains are >30 amino acids long and form non-globular elongated structures. Structure-based functionality of these repeated domains has been illustrated for tetratricopeptide (TPR) [40] and spectrin [41]. Specifically, some repeats serve as building blocks in superstructure and thus steric constraints limit the expansion of the repeated units [11].

The parameters used in this study for identification of TR-proteins in *N. vectensis *excluded most of the Pfam domains [42]. However, overall, the Pfam-defined repeated domains from *N. vectensis *exhibit a copy number similar to their mammalian counterparts (Figure 7). At present, the validated number of repeated TR elements in *N. vectensis *cannot be determined. We expect a fraction of them to be eliminated following an exhaustive transcriptomic study. On the other hand, TRs with low sequence identity (<80% amino acid identity with the consensus) were not considered in our study.

Currently, functional information for *N. vectensis *(i.e., GO annotations [43]) is very limited. Annotations that appears in >40 proteins are sparse and include 'Zinc ion binding' (46 proteins), 'G-protein coupled receptor protein signaling' (42 proteins), and 'Calcium binding' (45 proteins). While the overlap of *N. vectensis *TR-units with TR-units from Hydra is rather limited (Figure 6A), about 50 of the TR-units are shared only among Cnidaria representatives and are not detected in human or mouse (Additional file 3).

### Evolution dynamics - expansion and deletion of repeated units

Representative genomes from Porifera (sponges) have provided molecular information on the evolutionarily oldest, yet still extant, phylum. Analysis of the genomes of Demospongiae (*Suberites domuncula *and *Geodia cydonium*), Calcarea (*Sycon raphanus*), and Hexactinellida (*Aphrocallistes vastus*) revealed an increase in gene number due to a burst of gene duplication events. This process was accompanied by efficient domain shuffling. This gave rise to the evolution of new domains (i.e., adhesion molecules) and elements of adaptive immunity [23].

We have shown that the expansion and contraction of repeats carries the potential for an increase in functional variability. The large numbers of TR-segments and additional repeated units in *N. vectensis *proteome may have an impact on the fitness of *Nematostella *to cope with the environment. We noted a wide variation in the extent of repeats throughout the evolutionary tree (Table 2). Repeat expansion has shaped many families of proteins (e.g., leucine-rich repeats [33]). The mechanism that underlies the gain and loss of TRs (of the type and size discussed in this study) is most likely a non-reciprocal mode of recombination and gene conversion. Over evolutionary time, gene conversion within repeated sequences has been an important mode in shaping eukaryotic genomes [44]. It is likely that most TR-units in the representative organisms studied (Figure 6) have evolved independently. The TR-proteins that are shared between human, mouse, hydra, sponge, choanoflagellate, and sea anemone genomes serve as a basis to study the process of their evolution. Exciting examples for the importance of the actual numbers of TR-units within coding sequences are known from yeast to human [45,46].

## Conclusions

The proteome of *Nematostella vectensis *is uniquely characterized in terms of its tandemly repeated amino acid sequences. About 3000 TR-units from *N. vectensis *were identified, a number that exceeds all other multicellular organisms tested. Comparing TR-proteins to additional representatives of metazoan origin (Hydra and Monosiga)substantiated the uniqueness of the phenomenon in *N. vectensis *but also confirmed that TR-units are in general less conserved even among closely related organisms (human and mouse). We suggest that the TR-proteins present in early metazoan life may have served as a robust source for novel genes with previously overlooked characteristics. These TR-proteins have unique properties in term of amino acid composition, structural properties, and evolutionary dynamics. As such, they may have unexpectedly functioned as a distinctive and rich origin for gene novelty within the framework of animal evolution.

## Methods

### Data Sources

For the complete Nematostella proteome, we used UniProtKB [47] covering 24,906 proteins (UniProt version 14.0). For each predicted protein, the translated cDNA sequence was extracted from the complete genome of *N. vectensis *from the Joint Genome Institute (JGI). The cDNA sequences of all other organisms analyzed were extracted from Ensembl using BioMart and its script suite [48].

The data used for assessment of ORF properties and for measuring the DNA sequence divergence rate (Ka/Ks) excluded those proteins for which the amino acid sequence and its corresponding cDNA conflicted. 19,283 sequences from the JGI sea anemone genome project version 1.0 http://genome.jgi-psf.org/ are associated with their protein sequence. Each of the ORF sequences was matched with the cDNA sequence and genome positioning (when available). Analyses were performed on this proteome-genome matched set. The sequences of the TR-segments were extracted to create a TR-only collection. This set covers 40% (of the amino acids) of the original data presented by the *N. vectensis *JGI complete genome.

### Bioinformatics resources

For sequence comparison, we applied NCBI resources and tools including Gene [49], NCBI-BLAST2 ftp://ftp.ncbi.nlm.nih.gov/blast/ and the JGI genome browser http://genome.jgi-psf.org. Functional annotations are based on InterPro and Pfam assignments. Pfam annotations from StellaBase [42] include 7868 entries for the entire proteome. Using annotations from the UniProtKB cross-reference indicates that 68% of all predicted proteins from *N. vectensis *have at least one PfamA entry. InterPro version 20.0 [10] lists 18,469 entries, 252 of them assigned as 'repeats'. 49 InterPro entries have no Pfam support and were not further analyzed. The rest of the repeats (203 entries, covering 175,000 UniProt proteins) were analyzed according to InterPro, GO 'molecular function' and 'biological process' terms, and Enzyme Classification (EC), as obtained from the UniProtKB resource.

### Prediction methods and tools

#### Tandem repeats

The locations of TRs in the proteins and the transcripts were determined using the Xstream web tool [20]. The parameters of Xstream for protein are (i) TRs are >70% identical (ii) The minimal length of the repeated unit is 3 amino acids (iii) The minimal domain length (defined as the total length of the repeated units) is 10 (iv) the repeated unit appears at least 3 times (v) each repeat unit shares >80% identity to the consensus sequence; (vi) there are at most 3 gaps in the repeats. For nucleic acids, the parameters that define TRs are (i) TRs are >70% identical (ii) The unit is of a minimal length of 10 nucleotides (iii) the minimal domain length is 30 nucleotides. Parameters iv-vi are identical for both proteins and nucleic acids.

#### Divergence rate

To calculate synonymous (Ks) and asynonymous (Ka) substitution rates, we used the Ka/Ks Calculator developed by the Beijing Genomics Institute [50]. The Ka/Ks ratio compares the rates of asynonymous and synonymous substitutions. A ratio that is <1 indicates that asynonymous mutations tend to be removed from the population. Positive selection (i.e., adaptive evolution) generates a ratio >1. For Ka/Ks measurement, we used only the ~400 TR-proteins that have EST evidence. For each protein, the Ka/Ks ratio was calculated relative to the first repeat unit, which was used as a reference. TR-proteins that show a variation rate <0.05 and TRs that are very short were excluded from the analysis, leaving 94 sequences.

#### Amino Acid properties

Composition Profiler [51] was applied for the analysis of amino acid properties for the TR-proteins, where the basic concept is to compare the amino acid probabilities estimated from a protein sample relative to those estimated from a background sample. P-values are calculated based on the hypothesis that the amino acid composition of the samples comes from the same underlying distribution (corrected for multiple tests). We used a set of 500 randomly chosen non-repeat proteins from the *N. vectensis *proteome as a background for the composition profile of ORF +1, and another such set for the similarities between the compositions for all 3 ORFs. Note that the composition for ORF +1 was nearly identical as compared to either background.

### Statistical estimation and measurement

In order to estimate the statistical significance of the number of valid (with open reading frame) ARFs per protein, we used simulated protein data. To construct such a protein *in silico*, we created a randomized set of protein sequences and rejected sequences containing stop codons in the first frame, thus exclusively generating proteins for which ORF+1 is valid. Based on the length distribution of the TR segments of the 4337 TR-proteins taken from the actual data, we created 4337 such simulated proteins. For each such protein, we evaluated all possible reading frames, and tallied the appearance of stop codons.

## Abbreviations

TR: tandem repeat; ORF: open reading frame; ARF: alternative reading frame.

## Authors' contributions

GN performed the research and led the data compilation and all analysis. He participated in writing the manuscript. MF supported the bioinformatics statistical aspects and the structural analysis. ML initiated, guided the research, the analyses and drafted the manuscript.

## Supplementary Material

Additional file 1 **Variation rate for TR segments in 3 representative proteomes**. This file shows the variation rate for all TR-segments from human, *H. magnipapillata *and *N. vectensis*. The data are complementary to Figure 3C. Click here for file

Additional file 2 **Protein candidates for long ARFs**. This file shows the long ARFs that are >500 nucleotides. IDs are according to FilterModel IDs from JGI. Click here for file

Additional file 3 **Shared TR in proteomes**. This table shows the TR-segments that are shared between *H. magnipapillata *and *N. vectensis *and those shared between human, mouse, and *N. vectensis*. The data are complementary to Figure 6. Click here for file

Additional file 4 **TR-segments from *N. vectensis ***This file depicts examples of TR-segments from *N. vectensis*. The data are complementary to Table 2. Click here for file

Additional file 5 **InterPro repeats in *N. vectensis *and human proteomes**. This file shows all Pfam entries with >20 proteins from *N. vectensis *(A). A list of all Pfam repeats entries alongside with the number of proteins in *N. vectensis *are shown (B). The data are complementary to Figure 7. Click here for file

## References

[B1] Makalowski W Mitchell GA Labuda D Alu sequences in the coding regions of mRNA: a source of protein variability Trends Genet 1994 10 188 193 10.1016/0168-9525(94)90254-2 8073532

[B2] Zhang L Yuan D Yu S Li Z Cao Y Miao Z Qian H Tang K Preference of simple sequence repeats in coding and non-coding regions of Arabidopsis thaliana Bioinformatics 2004 20 1081 1086 10.1093/bioinformatics/bth043 14764542

[B3] Kashi Y King D Soller M Simple sequence repeats as a source of quantitative genetic variation Trends Genet 1997 13 74 78 10.1016/S0168-9525(97)01008-1 9055609

[B4] Alba MM Tompa P Veitia RA Amino acid repeats and the structure and evolution of proteins Genome Dyn 2007 3 119 130 full_text 18753788 10.1159/000107607

[B5] Ackermann M Chao L DNA sequences shaped by selection for stability PLoS Genet 2006 2 e22 10.1371/journal.pgen.0020022 16518467 PMC1378130

[B6] Loire E Praz F Higuet D Netter P Achaz G Hypermutability of genes in Homo sapiens due to the hosting of long mono-SSR Mol Biol Evol 2009 26 111 121 10.1093/molbev/msn230 18845548

[B7] Mularoni L Veitia RA Alba MM Highly constrained proteins contain an unexpectedly large number of amino acid tandem repeats Genomics 2007 89 316 325 10.1016/j.ygeno.2006.11.011 17196365

[B8] Bowater RP Wells RD The intrinsically unstable life of DNA triplet repeats associated with human hereditary disorders Prog Nucleic Acid Res Mol Biol 2001 66 159 202 full_text 11051764 10.1016/s0079-6603(00)66029-4

[B9] Frey S Richter RP Gorlich D FG-rich repeats of nuclear pore proteins form a three-dimensional meshwork with hydrogel-like properties Science 2006 314 815 817 10.1126/science.1132516 17082456

[B10] Mulder N Apweiler R InterPro and InterProScan: tools for protein sequence classification and comparison Methods Mol Biol 2007 396 59 70 full_text 18025686 10.1007/978-1-59745-515-2_5

[B11] Andrade MA Perez-Iratxeta C Ponting CP Protein repeats: structures, functions, and evolution J Struct Biol 2001 134 117 131 10.1006/jsbi.2001.4392 11551174

[B12] de la Fuente J Garcia-Garcia JC Barbet AF Blouin EF Kocan KM Adhesion of outer membrane proteins containing tandem repeats of Anaplasma and Ehrlichia species (Rickettsiales: Anaplasmataceae) to tick cells Vet Microbiol 2004 98 313 322 10.1016/j.vetmic.2003.11.001 15036540

[B13] Portugaly E Harel A Linial N Linial M EVEREST: automatic identification and classification of protein domains in all protein sequences BMC Bioinformatics 2006 7 277 10.1186/1471-2105-7-277 16749920 PMC1533870

[B14] Katti MV Sami-Subbu R Ranjekar PK Gupta VS Amino acid repeat patterns in protein sequences: their diversity and structural-functional implications Protein Sci 2000 9 1203 1209 10.1110/ps.9.6.1203 10892812 PMC2144659

[B15] Heringa J Detection of internal repeats: how common are they? Curr Opin Struct Biol 1998 8 338 345 10.1016/S0959-440X(98)80068-7 9666330

[B16] Heger A Holm L Rapid automatic detection and alignment of repeats in protein sequences Proteins 2000 41 224 237 10.1002/1097-0134(20001101)41:2<224::AID-PROT70>3.0.CO;2-Z 10966575

[B17] Biegert A Soding J De novo identification of highly diverged protein repeats by probabilistic consistency Bioinformatics 2008 24 807 814 10.1093/bioinformatics/btn039 18245125

[B18] Gruber M Soding J Lupas AN REPPER--repeats and their periodicities in fibrous proteins Nucleic Acids Res 2005 33 W239 243 10.1093/nar/gki405 15980460 PMC1160166

[B19] Karpenahalli MR Lupas AN Soding J TPRpred: a tool for prediction of TPR-, PPR- and SEL1-like repeats from protein sequences BMC Bioinformatics 2007 8 2 10.1186/1471-2105-8-2 17199898 PMC1774580

[B20] Newman AM Cooper JB XSTREAM: a practical algorithm for identification and architecture modeling of tandem repeats in protein sequences BMC Bioinformatics 2007 8 382 10.1186/1471-2105-8-382 17931424 PMC2233649

[B21] King N Westbrook MJ Young SL Kuo A Abedin M Chapman J Fairclough S Hellsten U Isogai Y Letunic I The genome of the choanoflagellate Monosiga brevicollis and the origin of metazoans Nature 2008 451 783 788 10.1038/nature06617 18273011 PMC2562698

[B22] Putnam NH Srivastava M Hellsten U Dirks B Chapman J Salamov A Terry A Shapiro H Lindquist E Kapitonov VV Sea anemone genome reveals ancestral eumetazoan gene repertoire and genomic organization Science 2007 317 86 94 10.1126/science.1139158 17615350

[B23] Muller WE Schroder HC Skorokhod A Bunz C Muller IM Grebenjuk VA Contribution of sponge genes to unravel the genome of the hypothetical ancestor of Metazoa (Urmetazoa) Gene 2001 276 161 173 10.1016/S0378-1119(01)00669-2 11591483

[B24] Hemmrich G Anokhin B Zacharias H Bosch TC Molecular phylogenetics in Hydra, a classical model in evolutionary developmental biology Mol Phylogenet Evol 2007 44 281 290 10.1016/j.ympev.2006.10.031 17174108

[B25] Philippe H Derelle R Lopez P Pick K Borchiellini C Boury-Esnault N Vacelet J Renard E Houliston E Queinnec E Phylogenomics revives traditional views on deep animal relationships Curr Biol 2009 19 706 712 10.1016/j.cub.2009.02.052 19345102

[B26] Darling JA Reitzel AR Burton PM Mazza ME Ryan JF Sullivan JC Finnerty JR Rising starlet: the starlet sea anemone, Nematostella vectensis Bioessays 2005 27 211 221 10.1002/bies.20181 15666346

[B27] Taylor MS Ponting CP Copley RR Occurrence and consequences of coding sequence insertions and deletions in Mammalian genomes Genome Res 2004 14 555 566 10.1101/gr.1977804 15059996 PMC383299

[B28] Kondrashov FA Rogozin IB Wolf YI Koonin EV Selection in the evolution of gene duplications Genome Biol 2002 3 RESEARCH0008 10.1186/gb-2002-3-2-research0008 11864370 PMC65685

[B29] Nematostella DOE Joint Genome Institute 2002 http://www.jgi.doe.gov/genome-projects

[B30] Finn RD Mistry J Schuster-Bockler B Griffiths-Jones S Hollich V Lassmann T Moxon S Marshall M Khanna A Durbin R Pfam: clans, web tools and services Nucleic Acids Res 2006 34 D247 251 10.1093/nar/gkj149 16381856 PMC1347511

[B31] Richard GF Kerrest A Dujon B Comparative genomics and molecular dynamics of DNA repeats in eukaryotes Microbiol Mol Biol Rev 2008 72 686 727 10.1128/MMBR.00011-08 19052325 PMC2593564

[B32] Hancock JM Simon M Simple sequence repeats in proteins and their significance for network evolution Gene 2005 345 113 118 10.1016/j.gene.2004.11.023 15716087

[B33] Marcotte EM Pellegrini M Yeates TO Eisenberg D A census of protein repeats J Mol Biol 1999 293 151 160 10.1006/jmbi.1999.3136 10512723

[B34] Gatherer D McEwan NR Phylogenetic differences in content and intensity of periodic proteins J Mol Evol 2005 60 447 461 10.1007/s00239-004-0189-2 15883880

[B35] Chung WY Wadhawan S Szklarczyk R Pond SK Nekrutenko A A first look at ARFome: dual-coding genes in mammalian genomes PLoS Comput Biol 2007 3 e91 10.1371/journal.pcbi.0030091 17511511 PMC1868773

[B36] Ikehara K Amada F Yoshida S Mikata Y Tanaka A A possible origin of newly-born bacterial genes: significance of GC-rich nonstop frame on antisense strand Nucleic Acids Res 1996 24 4249 4255 10.1093/nar/24.21.4249 8932380 PMC146247

[B37] Kloss E Courtemanche N Barrick D Repeat-protein folding: New insights into origins of cooperativity, stability, and topology Archives of Biochemistry and Biophysics 2008 469 83 99 10.1016/j.abb.2007.08.034 17963718 PMC2474553

[B38] Liu J Rost B Domains, motifs and clusters in the protein universe Curr Opin Chem Biol 2003 7 5 11 10.1016/S1367-5931(02)00003-0 12547420

[B39] Cole C Barber JD Barton GJ The Jpred 3 secondary structure prediction server Nucleic Acids Res 2008 36 W197 201 10.1093/nar/gkn238 18463136 PMC2447793

[B40] Ferreiro DU Walczak AM Komives EA Wolynes PG The energy landscapes of repeat-containing proteins: topology, cooperativity, and the folding funnels of one-dimensional architectures PLoS Comput Biol 2008 4 e1000070 10.1371/journal.pcbi.1000070 18483553 PMC2366061

[B41] Stabach PR Simonovic I Ranieri MA Aboodi MS Steitz TA Simonovic M Morrow JS The structure of the ankyrin-binding site of {beta}-spectrin reveals how tandem spectrin-repeats generate unique ligand-binding properties Blood 2009 113 5377 5384 10.1182/blood-2008-10-184291 19168783 PMC2689040

[B42] Sullivan JC Ryan JF Watson JA Webb J Mullikin JC Rokhsar D Finnerty JR StellaBase: the Nematostella vectensis Genomics Database Nucleic Acids Res 2006 34 D495 499 10.1093/nar/gkj020 16381919 PMC1347383

[B43] Harris MA Clark J Ireland A Lomax J Ashburner M Foulger R Eilbeck K Lewis S Marshall B Mungall C The Gene Ontology (GO) database and informatics resource Nucleic Acids Res 2004 32 D258 261 10.1093/nar/gkh066 14681407 PMC308770

[B44] Gangloff S Zou H Rothstein R Gene conversion plays the major role in controlling the stability of large tandem repeats in yeast EMBO J 1996 15 1715 1725 8612596 PMC450084

[B45] Verstrepen KJ Jansen A Lewitter F Fink GR Intragenic tandem repeats generate functional variability Nat Genet 2005 37 986 990 10.1038/ng1618 16086015 PMC1462868

[B46] Ding YC Chi HC Grady DL Morishima A Kidd JR Kidd KK Flodman P Spence MA Schuck S Swanson JM Evidence of positive selection acting at the human dopamine receptor D4 gene locus Proc Natl Acad Sci USA 2002 99 309 314 10.1073/pnas.012464099 11756666 PMC117557

[B47] Wu CH Apweiler R Bairoch A Natale DA Barker WC Boeckmann B Ferro S Gasteiger E Huang H Lopez R The Universal Protein Resource (UniProt): an expanding universe of protein information Nucleic Acids Res 2006 34 D187 191 10.1093/nar/gkj161 16381842 PMC1347523

[B48] Flicek P Aken BL Beal K Ballester B Caccamo M Chen Y Clarke L Coates G Cunningham F Cutts T Ensembl 2008 Nucleic Acids Res 2008 36 D707 714 10.1093/nar/gkm988 18000006 PMC2238821

[B49] McGinnis S Madden TL BLAST: at the core of a powerful and diverse set of sequence analysis tools Nucleic Acids Res 2004 32 W20 25 10.1093/nar/gkh435 15215342 PMC441573

[B50] Zhang Z Li J Zhao XQ Wang J Wong GK Yu J KaKs_Calculator: calculating Ka and Ks through model selection and model averaging Genomics Proteomics Bioinformatics 2006 4 259 263 10.1016/S1672-0229(07)60007-2 17531802 PMC5054075

[B51] Vacic V Uversky VN Dunker AK Lonardi S Composition Profiler: a tool for discovery and visualization of amino acid composition differences BMC Bioinformatics 2007 8 211 10.1186/1471-2105-8-211 17578581 PMC1914087

[B52] Dunker AK Lawson JD Brown CJ Williams RM Romero P Oh JS Oldfield CJ Campen AM Ratliff CM Hipps KW Intrinsically disordered protein J Mol Graph Model 2001 19 26 59 10.1016/S1093-3263(00)00138-8 11381529

