# Supplementary material for: Expansion of tandem repeats in sea anemone Nematostella vectensis proteome: A source for gene novelty?
Source: BMC Genomics. 2009 Dec 10;10:593. doi: 10.1186/1471-2164-10-593 (PMC2805694; doi:10.1186/1471-2164-10-593)
Supplement: Additional file 1 — Variation rate for TR segments in 3 representative proteomes. This file shows the variation rate for all TR-segments from human, H. magnipapillata and N. vectensis. The data are complementary to Figure 3C. [file 1471-2164-10-593-S1.doc]

**Additional file 1**

| **Variation Rate** | **Human** | **Hydra** | ***N. Vectensis*** |
| --- | --- | --- | --- |
| 0 | 229 | 161 | 235 |
| 0.02 | 18 | 121 | 465 |
| 0.04 | 71 | 101 | 560 |
| 0.06 | 140 | 101 | 595 |
| 0.08 | 283 | 141 | 557 |
| 0.10 | 155 | 89 | 516 |
| 0.12 | 255 | 123 | 426 |
| 0.14 | 303 | 81 | 325 |
| 0.16 | 366 | 87 | 278 |
| 0.18 | 467 | 126 | 286 |
| 0.2 | 346 | 76 | 133 |
|  |  |  |  |
| total | 2633 | 1207 | 4376 |
