# Supplementary material for: Expansion of tandem repeats in sea anemone Nematostella vectensis proteome: A source for gene novelty?
Source: BMC Genomics. 2009 Dec 10;10:593. doi: 10.1186/1471-2164-10-593 (PMC2805694; doi:10.1186/1471-2164-10-593)
Supplement: Additional file 2 — Protein candidates for long ARFs. This file shows the long ARFs that are >500 nucleotides. IDs are according to FilterModel IDs from JGI. [file 1471-2164-10-593-S2.doc]

**Additional file 2**

Protein candidates for long ARFs (>500 nucleotides).

|  | **JGI ID** | **Length (nt)** | **3 ARFs have >70% identity** |
| --- | --- | --- | --- |
| 1 | 203844* | 2118 |  |
| 2 | 11536 | 2022 |  |
| 3 | 205470 | 1818 | yes |
| 4 | 216439* | 1677 | yes |
| 5 | 163707* | 1596 |  |
| 6 | 218247* | 1287 |  |
| 7 | 94826* | 1158 | yes |
| 8 | 118590 | 1086 | yes |
| 9 | 212069* | 1050 | yes |
| 10 | 148203* | 1008 |  |
| 11 | 205426* | 996 | yes |
| 12 | 208037 | 966 | yes |
| 13 | 34040* | 927 | yes |
| 14 | 219578* | 921 |  |
| 15 | 238136* | 885 | yes |
| 16 | 83684* | 873 |  |
| 17 | 222181 | 873 |  |
| 18 | 212370 | 870 | yes |
| 19 | 91438* | 870 | yes |
| 20 | 219847* | 852 | yes |
| 21 | 105287 | 846 |  |
| 22 | 106408 | 843 |  |
| 23 | 12853 | 831 |  |
| 24 | 15029 | 816 |  |
| 25 | 241673 | 810 | yes |
| 26 | 11719* | 801 |  |
| 27 | 3524 | 795 | yes |
| 28 | 136624* | 795 | yes |
| 29 | 71671 | 771 | yes |
| 30 | 112046 | 762 |  |
| 31 | 61990* | 750 | yes |
| 32 | 8943* | 747 | yes |
| 33 | 132739 | 744 |  |
| 34 | 43221 | 738 |  |
| 35 | 125146* | 732 |  |
| 36 | 211473* | 723 | yes |
| 37 | 90013* | 708 | yes |
| 38 | 206598* | 708 |  |
| 39 | 4107 | 705 |  |
| 40 | 86910 | 705 |  |
| 41 | 45776* | 696 | yes |
| 42 | 123235* | 696 |  |
| 43 | 56070* | 693 |  |
| 44 | 109908 | 690 |  |
| 45 | 143825 | 684 |  |
| 46 | 143109 | 681 |  |
| 47 | 137801* | 678 |  |
| 48 | 103832* | 675 | yes |
| 49 | 97189 | 669 | yes |
| 50 | 124988* | 660 | yes |
| 51 | 222368* | 657 | yes |
| 52 | 70258 | 657 | yes |
| 53 | 72006* | 648 | yes |
| 54 | 102365 | 648 | yes |
| 55 | 3184* | 639 | yes |
| 56 | 212801 | 639 |  |
| 57 | 216282* | 636 | yes |
| 58 | 143700* | 636 |  |
| 49 | 107933 | 633 |  |
| 60 | 134239 | 624 |  |
| 61 | 139891* | 621 |  |
| 62 | 95866* | 621 | yes |
| 63 | 124851 | 609 |  |
| 64 | 7543 | 603 | yes |
| 65 | 119389* | 603 |  |
| 66 | 95164* | 600 | yes |
| 67 | 205728* | 597 | yes |
| 68 | 222797* | 594 |  |
| 69 | 148245* | 588 | yes |
| 70 | 37729* | 588 | yes |
| 71 | 198584 | 588 | yes |
| 72 | 31238* | 585 | yes |
| 73 | 47816* | 582 |  |
| 74 | 138253* | 582 |  |
| 75 | 102284* | 579 |  |
| 76 | 99782* | 576 | yes |
| 77 | 42981* | 576 |  |
| 78 | 1713 | 573 |  |
| 79 | 86324* | 567 |  |
| 80 | 209581* | 564 | yes |
| 81 | 118333 | 558 |  |
| 82 | 211730 | 552 |  |
| 83 | 119389* | 546 |  |
| 84 | 198584 | 546 | yes |
| 85 | 223105 | 546 |  |
| 86 | 207754 | 543 | yes |
| 87 | 114369 | 543 | yes |
| 88 | 102706* | 543 | yes |
| 89 | 2616 | 543 |  |
| 90 | 107063 | 540 | yes |
| 91 | 1374 | 540 |  |
| 92 | 116376 | 537 |  |
| 93 | 147894* | 534 |  |
| 94 | 87692* | 531 | yes |
| 95 | 125841* | 531 |  |
| 96 | 109376 | 531 |  |
| 97 | 13667* | 531 |  |
| 98 | 53835 | 528 | yes |
| 99 | 206388 | 525 | yes |
| 100 | 149307* | 525 |  |
| 101 | 217834 | 519 | yes |
| 102 | 212668* | 519 |  |
| 103 | 205426* | 513 | yes |
| 104 | 107063 | 513 | yes |
| 105 | 153874* | 513 | yes |
| 106 | 140652* | 501 | yes |
| 107 | 93900 | 501 | yes |

*Protein contains methionine

IDs are according to FilterModel IDs from JGI. This track combines gene models predicted by different methods and are selected on the basis of homology to known proteins and completeness. A total of 27,273 models are supported by *N. vectensis* genome assembly 1.0.
