# Supplementary material for: Expansion of tandem repeats in sea anemone Nematostella vectensis proteome: A source for gene novelty?
Source: BMC Genomics. 2009 Dec 10;10:593. doi: 10.1186/1471-2164-10-593 (PMC2805694; doi:10.1186/1471-2164-10-593)
Supplement: Additional file 3 — Shared TR in proteomes. This table shows the TR-segments that are shared between H. magnipapillata and N. vectensis and those shared between human, mouse, and N. vectensis. The data are complementary to Figure 6. [file 1471-2164-10-593-S3.doc]

| **Additional data file S3** - Shared TR in proteomes. | | | | | | | |
| --- | --- | --- | --- | --- | --- | --- | --- |
| **83 TR-segments in 82 proteins shared between hydra and *N. vectensis*** | | | | **64 TR-segments in 61 proteins – shared between human, mouse and *N. vectensis*** | | | |
| **Unirot ID** | **Positions** | **TR-Unit** | **Copy Number** | **Uniprot ID** | **Positions** | **TR-Unit** | **Copy Number** |
| **A7SUP6_NEMVE *** | 1-532 | 76 | 7 | **A7SUP6_NEMVE** | 1-532 | 76 | 7.00 |
| **A7RF47_NEMVE *** | 82-321 | 37 | 6.49 | **A7RF47_NEMVE** | 82-321 | 37 | 6.49 |
| A7SEJ5_NEMVE | 1779-1841 | 13 | 4.85 | A7SWT3_NEMVE | 1-355 | 28 | 12.68 |
| A8DUU5_NEMVE | 1-159 | 12 | 13.25 | **A7RVI6_NEMVE** | 177-215 | 11 | 3.27 |
| **A7RVI6_NEMVE** | 177-215 | 11 | 3.27 | **A7S9Q5_NEMVE** | 134-181 | 11 | 3.91 |
| **A7S9Q5_NEMVE** | 134-181 | 11 | 3.91 | A7RSK6_NEMVE | 9-62 | 10 | 5.40 |
| A7RSU6_NEMVE | 102-256 | 10 | 15.1 | **A7RTJ6_NEMVE** | 297-328 | 10 | 3.10 |
| **A7RTJ6_NEMVE** | 297-328 | 10 | 3.1 | **A7SMB0_NEMVE** | 87-120 | 10 | 3.20 |
| **A7SMB0_NEMVE** | 87-120 | 10 | 3.2 | **A7SMY8_NEMVE** | 272-312 | 10 | 4.10 |
| **A7SMY8_NEMVE** | 272-312 | 10 | 4.1 | A7SV34_NEMVE | 61-104 | 9 | 4.89 |
| A7SVL8_NEMVE | 1-49 | 10 | 4.9 | **A7RF99_NEMVE**** | 140-173 | 8 | 4.25 |
| A7SX39_NEMVE | 33-426 | 10 | 39.4 | A7RUE3_NEMVE | 247-276 | 8 | 3.75 |
| A7SP12_NEMVE | 1-157 | 9 | 17.44 | **A7SHB1_NEMVE** | 466-501 | 8 | 4.38 |
| A7RH26_NEMVE | 1-258 | 8 | 32.25 | A7RLH2_NEMVE | 201-230 | 7 | 4.29 |
| A7RPR2_NEMVE | 1-102 | 8 | 12.62 | **A7RLP1_NEMVE** | 175-215 | 7 | 5.57 |
| **A7SHB1_NEMVE** | 466-501 | 8 | 4.38 | A7RXZ8_NEMVE | 2-192 | 7 | 27.29 |
| A7T1W3_NEMVE | 356-387 | 8 | 4 | **A7RYJ0_NEMVE** | 357-408 | 7 | 7.43 |
| A7RFW7_NEMVE | 99-159 | 7 | 8.71 | A7S1Z4_NEMVE | 300-321 | 7 | 3.14 |
| A7RGG2_NEMVE | 323-441 | 7 | 17 | **A7S288_NEMVE** | 1099-1122 | 7 | 3.29 |
| A7RH87_NEMVE | 1-116 | 7 | 16.57 | **A7S6Z1_NEMVE** | 311-346 | 7 | 5.00 |
| **A7RLP1_NEMVE** | 175-215 | 7 | 5.57 | **A7S8F9_NEMVE** | 1603-1889 | 7 | 40.57 |
| A7RP57_NEMVE | 63-158 | 7 | 13.57 | A7S907_NEMVE | 596-619 | 7 | 3.43 |
| A7RQX7_NEMVE | 520-547 | 7 | 4 | **A7SAV5_NEMVE** | 59-82 | 7 | 3.43 |
| A7RSQ1_NEMVE | 173-206 | 7 | 4.86 | A7SAW4_NEMVE | 259-280 | 7 | 3.14 |
| **A7RYJ0_NEMVE** | 357-408 | 7 | 7.43 | A7SFB1_NEMVE | 1-102 | 7 | 14.57 |
| A7S0C8_NEMVE | 2-52 | 7 | 7.29 | A7SIX6_NEMVE | 136-198 | 7 | 9.00 |
| **A7S288_NEMVE** | 1099-1122 | 7 | 3.29 | **A7S6E5_NEMVE**** | 689-710 | 6 | 3.67 |
| **A7S6Z1_NEMVE** | 311-346 | 7 | 5 | **A7RF99_NEMVE**** | 334-348 | 5 | 3.00 |
| A7S782_NEMVE | 12-72 | 7 | 8.71 | A7RIX4_NEMVE | 1351-1370 | 5 | 3.80 |
| **A7S8F9_NEMVE** | 1603-1889 | 7 | 40.57 | A7RNZ0_NEMVE | 247-282 | 5 | 7.00 |
| A7S9F1_NEMVE | 1-190 | 7 | 27.14 | A7S1S3_NEMVE | 273-292 | 5 | 4.00 |
| **A7SAV5_NEMVE** | 59-82 | 7 | 3.43 | A7S554_NEMVE | 291-306 | 5 | 3.20 |
| A7SE69_NEMVE | 2-300 | 7 | 42.71 | A7S640_NEMVE | 416-439 | 5 | 4.60 |
| A7SFI5_NEMVE | 8-62 | 7 | 7.86 | **A7S6E5_NEMVE**** | 793-811 | 5 | 3.60 |
| A7SG95_NEMVE | 1-236 | 7 | 33.71 | A7S6E9_NEMVE | 50-69 | 5 | 4.00 |
| A7SP22_NEMVE | 154-175 | 7 | 3.14 | A7T070_NEMVE | 1231-1252 | 5 | 4.40 |
| A7SRS0_NEMVE | 70-108 | 7 | 5.57 | **A7RF99_NEMVE**** | 168-182 | 4 | 3.75 |
| A7ST36_NEMVE | 1-139 | 7 | 19.86 | **A7RGW1_NEMVE** | 279-335 | 4 | 14.25 |
| A7SUA2_NEMVE | 1-114 | 7 | 16.29 | **A7RMZ0_NEMVE** | 240-256 | 4 | 4.25 |
| A7SX78_NEMVE | 211-232 | 7 | 3.14 | A7RPV9_NEMVE** | 1-51 | 4 | 12.75 |
| A7SXV2_NEMVE | 1-361 | 7 | 50.71 | A7RPV9_NEMVE** | 46-143 | 4 | 24.50 |
| A7SY98_NEMVE | 1-278 | 7 | 39.57 | **A7RQL2_NEMVE** | 152-173 | 4 | 5.50 |
| A7T6D4_NEMVE | 107-128 | 7 | 3.14 | **A7RVR7_NEMVE** | 1436-1448 | 4 | 3.25 |
| A7T6I8_NEMVE | 9-32 | 7 | 3.43 | **A7RYS4_NEMVE** | 377-391 | 4 | 3.50 |
| A7RIX2_NEMVE | 1586-1633 | 6 | 8 | **A7RYT0_NEMVE** | 251-267 | 4 | 4.25 |
| A7S0W1_NEMVE | 10-30 | 6 | 3.5 | A7S0Z6_NEMVE | 13-81 | 4 | 17.00 |
| A7S3B6_NEMVE | 83-158 | 6 | 12.17 | A7S2A8_NEMVE | 71-93 | 4 | 5.75 |
| A7S9Q5_NEMVE | 7-31 | 6 | 4 | **A7S6E5_NEMVE**** | 1077-1092 | 4 | 4.00 |
| **A7RF99_NEMVE**** | 334-348 | 5 | 3 | **A7S6Q2_NEMVE** | 154-165 | 4 | 3.00 |
| A7RV09_NEMVE | 346-365 | 5 | 4 | **A7S9H3_NEMVE** | 172-191 | 4 | 4.75 |
| A7S896_NEMVE | 531-570 | 5 | 8 | **A7S9K8_NEMVE** | 239-265 | 4 | 6.00 |
| A7SAZ9_NEMVE | 319-335 | 5 | 3.4 | A7SGL4_NEMVE | 130-142 | 4 | 3.25 |
| A7SF69_NEMVE | 46-89 | 5 | 8.4 | **A7SI59_NEMVE** | 800-811 | 4 | 3.00 |
| **A7RF99_NEMVE**** | 168-182 | 4 | 3.75 | **A7SKU6_NEMVE** | 249-269 | 4 | 4.50 |
| **A7RGW1_NEMVE** | 279-335 | 4 | 14.25 | A7SNM7_NEMVE | 615-641 | 4 | 6.75 |
| **A7RMZ0_NEMVE** | 240-256 | 4 | 4.25 | **A7SPV2_NEMVE** | 33-52 | 4 | 5.00 |
| **A7RQL2_NEMVE** | 152-173 | 4 | 5.5 | **A7T285_NEMVE** | 164-196 | 4 | 7.50 |
| A7RUN7_NEMVE | 405-422 | 4 | 4.5 | **A7T3K9_NEMVE** | 110-148 | 4 | 9.75 |
| **A7RVR7_NEMVE** | 1436-1448 | 4 | 3.25 | **A7T4Y8_NEMVE** | 405-433 | 4 | 7.25 |
| **A7RYS4_NEMVE** | 377-391 | 4 | 3.5 | **A7RHP2_NEMVE** | 9-33 | 3 | 8.33 |
| **A7RYT0_NEMVE** | 251-267 | 4 | 4.25 | **A7RTY8_NEMVE** | 348-381 | 3 | 10.67 |
| A7S0A5_NEMVE | 800-969 | 4 | 42.5 | A7S5J0_NEMVE | 106-117 | 3 | 4.00 |
| A7S0Z6_NEMVE | 13-81 | 4 | 17 | A7SEJ9_NEMVE | 1967-1976 | 3 | 3.33 |
| **A7S6E5_NEMVE** | 1077-1092 | 4 | 4 | A7SIY4_NEMVE | 372-383 | 3 | 4.00 |
| **A7S6Q2_NEMVE** | 154-165 | 4 | 3 |  | | | |
| **A7S9H3_NEMVE** | 172-191 | 4 | 4.75 |
| **A7S9K8_NEMVE** | 239-265 | 4 | 6 |
| A7SB40_NEMVE | 366-406 | 4 | 9.75 |
| A7SGG1_NEMVE | 444-457 | 4 | 3.5 |
| **A7SI59_NEMVE** | 800-811 | 4 | 3 |
| **A7SKU6_NEMVE** | 249-269 | 4 | 4.5 |
| A7SLC5_NEMVE | 628-642 | 4 | 3.75 |
| A7SMS7_NEMVE | 752-767 | 4 | 4 |
| **A7SPV2_NEMVE** | 33-52 | 4 | 5 |
| **A7T285_NEMVE** | 164-196 | 4 | 7.5 |
| **A7T3K9_NEMVE** | 110-148 | 4 | 9.75 |
| **A7T4Y8_NEMVE** | 405-433 | 4 | 7.25 |
| A7T7Q7_NEMVE | 10-81 | 4 | 18 |
| **A7RHP2_NEMVE** | 9-33 | 3 | 8.33 |
| A7RHS7_NEMVE | 566-586 | 3 | 7 |
| **A7RTY8_NEMVE** | 348-381 | 3 | 10.67 |
| A7SGL5_NEMVE | 214-227 | 3 | 4.33 |
| A7TAX4_NEMVE | 276-288 | 3 | 4 |

* Proteins contain TRs that are discussed in Table 2. In bold are the 33 proteins from *N. vectensis* that are shared in all 4 organisms listed.

** marks proteins that appear with multiple TR-segments
