# Supplementary material for: Expansion of tandem repeats in sea anemone Nematostella vectensis proteome: A source for gene novelty?
Source: BMC Genomics. 2009 Dec 10;10:593. doi: 10.1186/1471-2164-10-593 (PMC2805694; doi:10.1186/1471-2164-10-593)
Supplement: Additional file 4 — TR-segments from N. vectensis This file depicts examples of TR-segments from N. vectensis. The data are complementary to Table 2. [file 1471-2164-10-593-S4.doc]

**Additional file 4.**

TR from *N. vectensis.*

7 sequences with a TR-unit of 114 are listed. Accession numbers are according to the UniProt database. For each sequence position, copy number and the variation rate (marked as Consensus Error) are shown.

**114 amino acids – 7 sequences (marked A-G)**

A. [tr|A7SI34|A7SI34_NEMVE Predicted protein (Frag...](http://jimcooperlab.mcdb.ucsb.edu/xstream/XSTREAMprog/output/XSTREAM_1243020935932_53c146519335f7732fc3245d8194bfb5.fasta_i0.7_g3_m3_L10_out_3.html#1853)

| Positions> | Period | Copy Number | Consensus Error |
| --- | --- | --- | --- |
| 1-1392 | 114 | 12.21 | 0.01 |

**TFIFASSSYSVEEDTGYVTVNITKIGTSDISLDVKLSTNNGTAFSPVDYTAMSDNMVTFLANEQSKLVNITINVDQTVENDEDFKALLSHTNADQVALGLLNTTTITIGNDDQA**

**TFSFASSSYSVEEDTGYVTVNITKIGTSDISLDVKLSTNNGTAFSPVDYTAMSDNMVTFLANEQSKLVNITINVDQTVENDEDFKALLSHTNADQVALGLLNTTTITIGNDDQA**

**TFSFASSSYSVEEDTGYVTVNITKIGTSDISLDVKLSTNNGTAFSPVDYTAMSDNMVTFLANEQSKLVNITINVDQTVENDEDFKALLSHTNADQVALGLLNTTTITIGNDDQA**

**TFSFASSSYSVEEDTGYVTVNITKIGTSDISLDVKLSTNNGTAFSPVDYTAMSDNMVTFLANEQSKLVNITINVDQTVENDEDFKALLSHTNADQVALGLLNTTTITIGNDDQA**

**TFSFASSSYSVEEDTGYVTVNITKIGTSDISLDVKLSTNNGTAFSPVDYTAMSDNMVTFLANEQSKLVNITINVDQTVENDEDFKALLSHTNADQVALGLLNTTTITIGNDDQA**

**TFSFASSSYSVEEDTGYVTVNITKTGTSDISLDVKLSTNNGTAFSPVDYTAMSDNMVTFLANEQSKLVNITINVDQTVENDEDFKALLSHTNADQVALGLLNTTTITIGNDDQA**

**TFSFASSSYSVEEDTGYVTVNITKTGTSDISLDVKLSTNNGTAFSPVDYTAMSDNSVTFLANEQSKLVNITINVDQTVENDEDFKALLSHANADQVALGLLNTTTITIGNDDQA**

**TFSFASSSYSVEEDTGYVTVNITKIGTSDISLDVKLSTNNGTAFSPVDYTAMSDNSVTFLANEQSKLVNITINVDQTVENDEDFKALLSHANADQVALGLLNTTTITIGNDDQA**

**TFSFASSSYSVEEDTGYVTVNITKIGTSDISLDVKLSTNNGTAFSPVDYTAMSDNSVTFLANEQSKLVNITINVDQTVENDEDFKALLSHTNADQVALGLLNTTTITIGNDDQA**

**TFSFASSSYSVEEDTGYVTVNITKTGTSDISLDVKLSTNNGTAFSPVDYTAMSDNMVTFLANEQSKLVNITINVDQTVENDEDFKALLSHTNADQVALGLLNTTTITIGNDDQA**

**TFSFASSSYSVEEDTGYVTVNITKIGTSDISLDVKLSTNNGTAFSPVDYTAMSDNMVTFLANEQSKLVNITINVDQTVENDEDFKALLSHTNADQVALGLLNTTTITIGNDDQA**

**TFSFASSSYSVEEDTGYVTVNITKTGTSDISLDVKLSTNNGTAFSPVDYTAMSDNGVTFLANEQSKLVNITINVDKTVENNEDFKALLSHTNADQVALGLLNTTTITIGNDDQA**

**IFSFASSSYSVEEDTGYVTLNITK**

==================================================================================================================

**TFSFASSSYSVEEDTGYVTVNITKIGTSDISLDVKLSTNNGTAFSPVDYTAMSDNMVTFLANEQSKLVNITINVDQTVENDEDFKALLSHTNADQVALGLLNTTTITIGNDDQA**

: : : : : : : :

B. [tr|A7SVI8|A7SVI8_NEMVE Predicted protein (Frag...](http://jimcooperlab.mcdb.ucsb.edu/xstream/XSTREAMprog/output/XSTREAM_1243020935932_53c146519335f7732fc3245d8194bfb5.fasta_i0.7_g3_m3_L10_out_3.html#2557)

| Positions> | Period | Copy Number | Consensus Error |
| --- | --- | --- | --- |
| 138-742 | 114 | 5.31 | 0.00 |

**ATFSFASSSYSVEEDTGYVTVNITKIGTSDISLDVKLSTNNGTAFSPVDYTAMSDNMVTFLANEQSKLVNITINVDQTVENDEDFKALLSHTNADQVALGLLNTTTITIGNDDQ**

**ATFSFASSSYSVEEDTGYVTVNITKIGTSDISLDVKLSTNNGTAFSPVDYTAISDNMVTFLANEQSKLVNITINVDQTVENDEDFKALLSHTNADQVALGLLNTTTITIGNDDQ**

**ATFSFASSSYSVEEDTGYVTVNITKIGTSDISLDVKLSTNNGTAFSPVDYTAMSDNMVTFLANEQSKLVNITINVDQTVENDEDFKALLSHTNADQVALGLLNTTTITIGNDDQ**

**ATFSFASSSYSVEEDTGYVTVNITKIGTSDISLDVKLSTNNGTAFSPVDYTAISDNMVTFLANEQSKLVNITINVDQTVENDEDFKALLSHTNADQVALGLLNTTTITIGNDDQ**

**ATFSFASSSYSVEEDTGYVTVNITKIGTSDISLDVKLSTNNGTAFSPVDYTAMSDNMVTFLANEQSKLVNITINVDQTVENDEDFKALLSHTNADQVALGLLNTTTITIGNDDQ**

**ATFSFASSSYSVEEDTGYVTVNITKIGTSDISLDV**

==================================================================================================================

**ATFSFASSSYSVEEDTGYVTVNITKIGTSDISLDVKLSTNNGTAFSPVDYTAMSDNMVTFLANEQSKLVNITINVDQTVENDEDFKALLSHTNADQVALGLLNTTTITIGNDDQ** :

C. [tr|A7SVI9|A7SVI9_NEMVE Predicted protein OS=Ne...](http://jimcooperlab.mcdb.ucsb.edu/xstream/XSTREAMprog/output/XSTREAM_1243020935932_53c146519335f7732fc3245d8194bfb5.fasta_i0.7_g3_m3_L10_out_3.html#2558)

| Positions> | Period | Copy Number | Consensus Error |
| --- | --- | --- | --- |
| 3-4112 | 114 | 36.05 | 0.04 |

**IAA--FGIAME-I-YQRK--CSRYNTIYV----YL-----T-------TFSFASSSYSVEEDTGYVT--VNITKTGTSDISLDVKLSTNNGTAFSPVDYTAMSDNSVTFLANEQSKLVNIT**

**I-N--VDQTVEND-EDFKALLSHTNADQV-ALGLLNTTTITIGNDDQATFSFASSSYSVEEDTGYVT--VNITKTGTSDISLDVKLSTNNGTAFSPVDYTAMSDNSVTFLANEQSKLVNIT**

**I-N--VDQTVEND-EDFKALLSHTNADQV-ALGLLNTTTITIGNDDQATFSFASSSYSVEEDTGYVT--VNITKTGTSDISLDVKLSTNNGTAFSPVDYTAMSDNSVTFLANEQSKLVNIT**

**I-N--VDQTVEND-EDFKALLSHTNADQV-ALGLLNTTTITIGNDDQATFSFASSSYSVEEDTGYVT--VNITKTGTSDISLDVKLSTNNGTAFSPVDYTAMSDNSVTFLANEQSKLVNIT**

**I-N--VDQTVEND-EDFKALLSHTNADQV-ALGLLNTTTITIGNDDQATFSFASPSYSVEEDTGYVT--VNITKTGTSDIYLDVKLSTNNGTAFSPVDYTAMSDNSVTFLANEQSKLVNIT**

**I-N--VDQTVEND-EDFKALLSHTNADQV-ALGLLNTTTITIGNDDQATFSFASSSYSVEEDTGYVT--VNITKTGTSDISLDVKLSTNNGTAFSPVDYTAMSDNSVTFLANEQSKLVNIT**

**I-N--VDQTVEND-EDFKALLSHTNADQV-ALGLLNTTTITIGNDDQATFSFASSSYSVEEDTGYVT--VNITKTGTSDINLDVKLSTNNGTAFSPVDYTAMSDNSVTFLANEQSKLVNIT**

**I-N--VDQTVEND-EDFKALLSHTNADQV-ALGLLNTTTITIGNDDRATFSFASSSYSVEEDTGYVT--VNITKTGTSDIHLDVKLSTNNGSAFSPVDYTAMSDNSVTFLANEQSKLVNIT**

**I-N--VDQTVEND-EDFKALLSHTNGDQV-ALGLLNTTTITIGNDDQATFSFASSSYSVEEDTGYVT--VNITKTGTSDISLDVKLSTNNGTAFSPVDYTAMSDNSVTFLANEQSKLVNIT**

**I-N--VDQTVEND-EDFKALLSHTNADQV-ALGLLNTTTITIGNDDQATFSFASSSYSVEEDTGYVT--VNITKTGTSDISLDVKLSTNNGTAFSPVDYTAMSDNSVTFLANEQSKLVNIT**

**I-N--VDQTVEND-EDFKALLSHTNADQV-ALGLLNTTTITIGNDDQATFSFVSSSYSVEEDTGYVS--VNITKTGTSDISLDVKLSTNNGTAFSPVDYTAMSDNSVTFLANEQSKLVNIT**

**I-N--VDQTVEND-EDFKALLSHTNADQV-ALGLLNTTTITIGNDDQATFSFASSSYSVEEDTGYVT--VNITKTGTSDISLDVKLSTNNGTAFSPVDYTAMSDNSVTFLANEQSKLVNIT**

**I-N--VDQTVEND-EDFKALLSHTNADQV-ALGLLNTTTITIGNDDQATFSFVSSSYSVEEDTGYVT--VNITKTGTSDISLDVKLSTNNGTAFSPVDYTAMSDNSVTFLANEQSKLVNIT**

**I-N--VDQTVEND-EDFKALLSYTNADQV-ALGLLNTTTITIGNDDQATFSFASSSFSVEEDTGYVT--VNITKTGTSDISLDVKLSTNNGTAFSPVDYTAMSDNSVTFLANEQSKLVNIT**

**I-N--VDQTVEND-EDFKALLSHTNADQV-ALGLLNTTTITIGNDDQATFSFVSSSYSVEEDTGYVT--VNITKTGTSDISLDVKLSTNNGTAFSPVDYTAMSDNSLTFLANEQSKLVNIT**

**I-N--VDQTVEND-EDFKALLSHTNADKV-ALGLLNTTTITIGNDDQ-----------VEEDTGYVT--VNITKTGTSDISLDVKLSTNNGTAFSPVDYTAMSDNSVTFLANEQSKLVNIT**

**I-N--VDQTVEND-EDFKALLSHTNADQV-ALGLLNTTTITIGNDDQATFSFASSSYSVDEDTGYVT--VNITKTGTSDISLDVKLSTNNGTAFSPVDYTAMSDNSVTFLANEQSKLVNIT**

**I-N--VDQTVEND-EDFKALLSHTNADQV-ALGLLNTTTITIGNDDQATFSFASSSYSVEEDTGYVT--VNITKTGTSDISLDVKLSTNNGTAFSPVDYTAMSDNSVTFLANEQSKLVNIT**

**I-N--VDQTVEND-EDFKALLSHTNADQV-ALGLLNTTTITIGNDDQATFSFASSSYSVEEDTGYVT--VNITKTGTSDISLDVKLSTNNGTAFSPVDYTAMSDNSVTFLANEQSKLVNIT**

**I-N--VDQTVENE-EDFKALLSHTNADQV-ALGLLNTTTITIGNDDQATFSFASPSYSVEEDTGYVT--VNITKTGTSDISLDVKLSTNNGTAFSPVDYTAMSDNSVTFLANEQSKLVNIT**

**I-N--VDQTVEND-EDFKALLSHTNADQV-ALGLLNTTTITIGNDDQATFSFASPSYSVEEDTGYVT--VNITKTGTSDIHLDVKLSTNNGTAFSPVDYTAMSDNSVTFLANEQSKLVNIT**

**I-N--VDQTVEND-EDFKALLSHTNADQV-ALGLLNTTTITIGNDDQATFSFASPSYSVEEDTGYVT--VNITKTGTSDI----HL-----------------D-------------VN--**

**K-NDIVD-IRPNDTKDAEGNPSEPPADIVENL-CPN-ECSNHGN-----CS--NSTCICEE--G-FTSLDCSLSINT-VPEL-LGLSTNNGTAFSPVDYTAMSDNSVTFLANEQSKLVNIT**

**I-N--VDQTVEND-EDFKALLSHTNADQV-ALGLLNTTTITIGNDDQATFSFASSSYSVEEDTGYVT--VNITKTGTSDISLDVKLSTNNGTAFSPVDYTAMSDNSVTFLANEQSKLVNIT**

**I-N--VDQTVEND-EDFKALLSHTNADQV-ALGLLNTTTITIGNDDQATFSFASSSYSVEEDTGYVT--VNITKTGTSDISLDVKLSTNNGTAFSPVDYTAMSDNSVTFLANEQSKLVNIT**

**I-N--VDQTVEND-EDFKALLSHTNADQV-ALGLLNTTTITIGNDDQATFSFASSSYSVEEDTGYVT--VNITKTGTSDISLDVKLSTNNGTAFSPVDYTAMSDNSVTFLANEQSKLVNIT**

**I-N--VDQTVEND-EDFKALLSHTNADQV-ALGLLNTTTITIGNDDQATFSFASPSYSVEEDTGYVT--VNITKTGTSDIHLDVKLSTNNGTAFSPVDYTAMSDNSVTFLANEQSKLVNIT**

**I-N--VDQTVEND-EDFKALLSHTNADQV-ALGLLNTTTITIGNDDQATFSFASPSYSVEEDTGYVT--VNITKTGTSDIHLDVKLSTNNGTAFSPVDYTAMSDNSVTFLANEQSKLVNIT**

**I-N--VDQTVEND-EDFKALLSHTNADQV-ALGLLNTTTITIGNDDQATFSFASSSYSVEEDTGYVT--VNITKTGTSDISLDVKLSTNNGTAFSPVDYTAMSDNSVTFLANEQSKLVNIT**

**I-N--VDQTVENE-EDFKALLSHTNADQV-ALGLLNTTTITIGNDDQATFSFASSSYSVEEDTGYVT--VNITKTGTSDISLDVQLSTNNGTAFSPVDYTAMSDNSVTFLANEQSKLVNIT**

**I-N--VDQTVENE-EDFKALLSHTNADQV-ALGLLNTTTITIGNDDQATFSFASSSYSVEEDTGYVT--VNITKTGTSDIHLDVKLSTNNGTAFSPVDYTAMSDNSVTFLANEQSKLVNIT**

**I-N--VDQTVEND-EDFKALLSHTNADQV-ALGLLNTTTITIGNDDQATFSFASPSYSVEEDTGYVT--VNITKTGTSEIHLDVKLSTNNGTAFSPVDYTAMSDNSVTFLANEQSKLVNIT**

**I-N--VDQTVEND-EDFKALLSHTNADQV-ALGLLNTTTITIGNDDQATFSFASPSYSVEEDTGYVT--VNITKTGTSEIHLDVKLSTNNGTAFSPVDYTAMSDNSVTFLANEQSKLVNIT**

**I-N--VDQTVEND-EDFKALLSHTNADQV-ALGLLNTTTITIGNDDQATFSFVSSSYSVEEDTGYVT--VNITKTGTSDISLDVKLSTNNGTAFSPVDYTAMSDNSVTFLANEQSKLVNIT**

**I-N--VDQTVEND-EDFKALLSHTNADQV-ALGLLNTTTITIGNDDQATFSFASSSYSVEEDTGYVT--VNITKTGTSDISLDVKLSTNNGTAFSPVDYTAMSDNSVTFLANEQSKLVNIT**

**I-N--VDQTVEND-EDFKALLSHTNADQV-ALGLLNTTTITIGNDDQATFSFASSSYSVEEDTGYVT--VNITKTGTSEISLDVKLSTNNGTAFSPVDYTAMSDNSVTFLANEQSKLVNIT**

**I-N--VDQTVENN-EDFKALLSHTNADQV-ALGLLNTTTITIGNDDQAIFSFASSSYSVEEDTGYVT--VNITKTGSSDIPLNVIL**

=========================================================================================================================

**I-N--VDQTVEND-EDFKALLSHTNADQV-ALGLLNTTTITIGNDDQATFSFASSSYSVEEDTGYVT--VNITKTGTSDISLDVKLSTNNGTAFSPVDYTAMSDNSVTFLANEQSKLVNIT**

::::::::::::::::::::: :::::: ::::::::::::::::::::::::::::::: :: ::::::::::::::::::::: ::::::::::::::::: ::::::::::::: ::

D. [tr|A7SVJ1|A7SVJ1_NEMVE Predicted protein OS=Ne...](http://jimcooperlab.mcdb.ucsb.edu/xstream/XSTREAMprog/output/XSTREAM_1243020935932_53c146519335f7732fc3245d8194bfb5.fasta_i0.7_g3_m3_L10_out_3.html#2560)

| Positions> | Period | Copy Number | Consensus Error |
| --- | --- | --- | --- |
| 87-433 | 114 | 3.04 | 0.10 |

**ANETS--YNFTVNINDDVIVENTESFFVQL--TTTD----GNINITQPNITVSI-TDNDKATFSFASSSYSVEEDTGYVTVNITKTGTSDINLDVKLSTNNGTAFSPVDYTAMSDNSVTFL**

**ANEQSKLVNIT--INVDQTVENDEDFKALLSHTNADQVALGLLN-T-TTIT--IGND-DQATFSFASPSYSVEEDTGYVTVNITKTGTSDIHLDVKLSTNNGTAFSPVDYTAMSDNSVTFL**

**ANEQSKLVNIT--INVDQTVENDEDFKALLSHTNADQVALGLLN-T-TTIT--IGND-DQATFSFASSSYSVEEDTGYVTVNITKTGTSDIHLDVKLSTNNGTAFSPVDYTAMSDNSVTFL**

**ANEQSKL**

=========================================================================================================================

**ANEQSKLVNIT--INVDQTVENDEDFKALLSHTNADQVALGLLN-T-TTIT--IGND-DQATFSFASSSYSVEEDTGYVTVNITKTGTSDIHLDVKLSTNNGTAFSPVDYTAMSDNSVTFL**

: ::: : :: : :: : : ::: :: :: :::: :: : ::: :: :: : : : :

E. [tr|A7TAK6|A7TAK6_NEMVE Predicted protein (Frag...](http://jimcooperlab.mcdb.ucsb.edu/xstream/XSTREAMprog/output/XSTREAM_1243020935932_53c146519335f7732fc3245d8194bfb5.fasta_i0.7_g3_m3_L10_out_3.html#3590)

| Positions> | Period | Copy Number | Consensus Error |
| --- | --- | --- | --- |
| 1-467 | 114 | 4.11 | 0.06 |

**ASSSYSVEEDT-GYVTVNITKIGTSDISLDVKLSTNNGTAFSPVDYTAMSDNMVTFLANEQSKLVNITINVDQTVENDEDFKALLSHTNADQVALGLLNTTTITIGNDDQATFSF**

**ASSSYSVEEDT-GYVTVNITKTGASDISLDVKLSTNNGTAFSPVDYTAMSDNMVTFLANEQSKLVNITINVDQTVENDEDFKALLSHTNADQVALGLLNTTTITIGNDDQ----V**

**A---Y-------CMV-FN----NHS-FALCLRLSTNNGTAFSPVDYTAMSDNMVTFLANEQSKLVNITINVDQTVENDEDFKALLSHTNADQVALGLLNTTTITIGNDDQATFSF**

**VSSSYSVEEDT-GYVTVNITKTGTSDISLDVKLSTNNGTAFSPVDYTAMSDNMVTFLANEQSKLVNITINVDQTVENDEDFKALLSHTNADQVALGLLNTTTITIGNDDQATFSF**

**ASSSYSVEEDTTGYVTVNITKTGTSDISLDV**

===================================================================================================================

**ASSSYSVEEDT-GYVTVNITKTGTSDISLDVKLSTNNGTAFSPVDYTAMSDNMVTFLANEQSKLVNITINVDQTVENDEDFKALLSHTNADQVALGLLNTTTITIGNDDQATFSF**

:::: ::::::::: :: :::::: ::: ::: :::::

F. [tr|A8DUR2|A8DUR2_NEMVE Predicted protein (Frag...](http://jimcooperlab.mcdb.ucsb.edu/xstream/XSTREAMprog/output/XSTREAM_1243020935932_53c146519335f7732fc3245d8194bfb5.fasta_i0.7_g3_m3_L10_out_3.html#3779)

| Positions> | Period | Copy Number | Consensus Error |
| --- | --- | --- | --- |
| 1-398 | 114 | 3.49 | 0.00 |

**TFSFASSSYSVEEDTGYVTVNITKIGTSDISLDVKLSTNNGTAFSPVDYTAMFDNMVTFLANEQSKLVNITINVDQTVENDEDFKALLSHTNADQVALGLLNTTTITIGNDDQA**

**TFSFASSSYSVEEDTGYVTVNITKIGTSDISLDVKLSTNNGTAFSPVDYTAMSDNMVTFLANEQSKLVNITINVDQTVENDEDFKALLSHTNADQVALGLLNTTTITIGNDDQA**

**TFSFASSSYSVEEDTGYVTVNITKIGTSDISLDVKLSTNNGTAFSPVDYTAMSDNMVTFLANEQSKLVNITINVDQTVENDEDFKALLSHTNADQVALGLLNTTTITIGNDDQA**

**TFSFASSSYSVEEDTGYVTVNITKIGTSDISLDVKLSTNNGTAFSPVDYTAMSDNM**

==================================================================================================================

**TFSFASSSYSVEEDTGYVTVNITKIGTSDISLDVKLSTNNGTAFSPVDYTAMSDNMVTFLANEQSKLVNITINVDQTVENDEDFKALLSHTNADQVALGLLNTTTITIGNDDQA**

:

G. [tr|A8DUS0|A8DUS0_NEMVE Predicted protein (Frag...](http://jimcooperlab.mcdb.ucsb.edu/xstream/XSTREAMprog/output/XSTREAM_1243020935932_53c146519335f7732fc3245d8194bfb5.fasta_i0.7_g3_m3_L10_out_3.html#3780)

| Positions> | Period | Copy Number | Consensus Error |
| --- | --- | --- | --- |
| 1-607 | 114 | 5.32 | 0.01 |

**TVENDEDFKALLSHTNADQVALGLLNTTTITIGNDDQATFSFASSSYSVEEDTGYVTVNITKTGTSDISLDVKLSTNNGTAFSPVDYTAMSDNMVTFLANEQSKLVNITINVDQ**

**TVENDEDFKALLSHTNADQVALGLLNTTTITIGNDDQATFSFASSSYSVEEDTGYVTVNITKIGTSDISLDVKLSTNNGTAFSPVDYTAMSDNMVTFLANEQSKLVNITINVDQ**

**TVENDEDFKALLSHTKADQVALGLLNTTTITIGNDDQATFSFASSSYSVEEDTGYVTVNITKTGTSDISLDVKLSTNNGTAFSPVDYTAMSDNMVTFLANEQSKLVNITINVDQ**

**TVENDEDFKALLSHTNADQVALGLLNTTTITIGNDDQATFSFASSSYSVEEDTGYVTVNITKIGTSDISLDVKLSTNNGTAFSPVDYTAMSDNMVTFLANEQSKLVNITINVDQ**

**TVENDEDFKALLSHTNADQVALGLLNTTTITIGNDDQATFSFASSSYSVEEDTGYVTVNITKIGTSDISVDVKLSTNNGTAFSPVDYTAMSDNMVTFLANEQSKLVNITINVDQ**

**TVENDEDFKALLSHTNADQVALGLLNTTTITIGNDDQ**

==================================================================================================================

**TVENDEDFKALLSHTNADQVALGLLNTTTITIGNDDQATFSFASSSYSVEEDTGYVTVNITKIGTSDISLDVKLSTNNGTAFSPVDYTAMSDNMVTFLANEQSKLVNITINVDQ**
