# Supplementary material for: Expansion of tandem repeats in sea anemone Nematostella vectensis proteome: A source for gene novelty?
Source: BMC Genomics. 2009 Dec 10;10:593. doi: 10.1186/1471-2164-10-593 (PMC2805694; doi:10.1186/1471-2164-10-593)
Supplement: Additional file 5 — InterPro repeats in N. vectensis and human proteomes. This file shows all Pfam entries with >20 proteins from N. vectensis (A). A list of all Pfam repeats entries alongside with the number of proteins in N. vectensis are shown (B). The data are complementary to Figure 7. [file 1471-2164-10-593-S5.doc]

**Additional file 5.**

InterPro repeats in *N. vectensis* and human proteomes.

InterPro repeats in *N. vectensis* (N.v.) and Human proteomes. All 252 repeats from InterPro were analyzed and mapped to Pfam repeated domains. A. Pfam entries with >20 proteins from *N. vectensis* are listed. B. List of all Pfam entries for repeats and the number of proteins in UniProt and in *N. vectensis*.

| **A.**  **Pfam entry** | **Pfam ID** | **N.v. proteins** | **N.v.**  **TR (n)** | **Human proteins** | **Human TR (n)** |
| --- | --- | --- | --- | --- | --- |
| Low-density lipoprotein receptor B | PF00058 | 22 | 4.7 | 44 | 11.6 |
| MORN motif | PF02493 | 25 | 5.6 | 40 | 5.4 |
| Armadillo | PF00514 | 25 | 3.8 | 77 | 4.6 |
| Low density lipoprotein-receptor, A | PF00057 | 32 | 3.8 | 103 | 5.1 |
| Tetratricopeptide TPR2 | PF07719 | 33 | 1.6 | 54 | 1.1 |
| NHL repeat | PF01436 | 37 | 3.9 | 27 | 3.4 |
| HEAT | PF02985 | 41 | 2.1 | 116 | 2.3 |
| Filamin/ABP280 repeat-like | PF00630 | 41 | 2.0 | 33 | 11.0 |
| Mitochondrial substrate/solute carrier | PF00153 | 54 | 2.7 | 120 | 2.6 |
| Kelch repeat type 1 | PF01344 | 72 | 4.0 | 166 | 4.1 |
| Collagen triple helix repeat | PF01391 | 76 | 3.1 | 206 | 6.8 |
| Leucine-rich repeat | PF00560 | 95 | 2.9 | 437 | 4.0 |
| Thrombospondin, type 1 repeat | PF00090 | 113 | 2.3 | 146 | 3.4 |
| Tetratricopeptide TPR-1 | PF00515 | 129 | 6.1 | 259 | 3.7 |
| Ankyrin | PF00023 | 179 | 3.2 | 616 | 5.4 |
| WD40 repeat, subgroup | PF00400 | 241 | 3.6 | 609 | 3.6 |

| **B.**  **InterPro** | **Name** | **Pfam** | **Short Abbrev.** | **# of proteins** | **# of N.v. proteins** |
| --- | --- | --- | --- | --- | --- |
| IPR000033 | Low-density lipoprotein receptor, YWTD | PF00058 | Low-dens YWTD | 596 | 22 |
| IPR000127 | Ubiquitin-activating enzyme repeat | PF02134 | UBACT | 528 | 4 |
| IPR000225 | Armadillo | PF00514 | Arm | 1735 | 25 |
| IPR000354 | Involucrin repeat | PF00904 | Involucrin | 40 | 1 |
| IPR000357 | HEAT | PF02985 | HEAT | 3685 | 41 |
| IPR000408 | Regulator of chromosome condens, RCC1 | PF00415 | RCC1 | 1104 | 19 |
| IPR000479 | Cation-independent mannose-6-phosphate | PF00878 | CIMR | 83 | 1 |
| IPR000547 | Clathrin, heavy chain/VPS, 7-fold repeat | PF00637 | Clathrin | 338 | 2 |
| IPR000557 | Calponin repeat | PF00402 | Calponin | 228 | 5 |
| IPR000684 | RNA polymerase II, heptapeptide repeat, | PF05001 | RNA_pol_Rpb1_R | 168 | 2 |
| IPR000861 | HR1-like rho-binding repeat | PF02185 | HR1 | 229 | 2 |
| IPR000884 | Thrombospondin, type 1 repeat | PF00090 | TSP_1 | 2056 | 113 |
| IPR000900 | Nebulin 35 residue motif | PF00880 | Nebulin | 146 | 3 |
| IPR001258 | NHL repeat | PF01436 | NHL | 2208 | 37 |
| IPR001313 | Pumilio RNA-binding region | PF00806 | PUF | 752 | 2 |
| IPR001330 | Prenyltransferase/squalene oxidase | PF00432 | Prenyltrans | 1238 | 2 |
| IPR001370 | Proteinase inhibitor I32, inhibitor of apoptosis | PF00653 | BIR | 505 | 3 |
| IPR001440 | Tetratricopeptide TPR-1 | PF00515 | TPR_1 | 16697 | 129 |
| IPR001442 | Type 4 procollagen, C-terminal repeat | PF01413 | C4 | 163 | 4 |
| IPR001451 | Bacterial transferase hexapeptide repeat | PF00132 | Hexapep | 11106 | 15 |
| IPR001611 | Leucine-rich repeat | PF00560 | LRR_1 | 19516 | 95 |
| IPR001646 | Pentapeptide repeat | PF00805 | Pentapeptide | 2644 | 3 |
| IPR001893 | Cysteine rich repeat | PF00839 | Cys_rich_FGFR | 65 | 1 |
| IPR002015 | Proteasome/cyclosome, regulatory subunit | PF01851 | PC_rep | 441 | 5 |
| IPR002017 | Spectrin repeat | PF00435 | Spectrin | 838 | 18 |
| IPR002088 | Protein prenyltransferase, alpha subunit | PF01239 | PPTA | 344 | 3 |
| IPR002110 | Ankyrin | PF00023 | Ank | 16638 | 179 |
| IPR002165 | Plexin | PF01437 | PSI | 633 | 5 |
| IPR002172 | Low density lipoprotein-receptor, cysteine-rich | PF00057 | Ldl_recept_a | 1652 | 32 |
| IPR002372 | Pyrrolo-quinoline quinone repeat | PF01011 | PQQ | 1683 | 2 |
| IPR002499 | Major vault protein, N-terminal | PF01505 | Vault | 61 | 2 |
| IPR002515 | Zinc finger, C2HC-type | PF01530 | zf-C2HC | 173 | 3 |
| IPR002860 | BNR repeat | PF02012 | BNR | 437 | 1 |
| IPR002885 | Pentatricopeptide repeat | PF01535 | PPR | 5162 | 5 |
| IPR003107 | RNA-processing protein, HAT helix | PF02184 | HAT | 209 | 1 |
| IPR003134 | Hs1/Cortactin | PF02218 | HS1_rep | 75 | 1 |
| IPR003367 | Thrombospondin, type 3-like repeat | PF02412 | TSP_3 | 817 | 4 |
| IPR003368 | Polymorphic membrane protein, Chlamydia | PF02415 | Chlam_PMP | 923 | 6 |
| IPR003409 | MORN motif | PF02493 | MORN | 1652 | 25 |
| IPR003822 | Paired amphipathic helix | PF02671 | PAH | 231 | 1 |
| IPR003883 | Extensin-like protein | PF02095 | Extensin_1 | 75 | 4 |
| IPR004018 | RPEL repeat | PF02755 | RPEL | 151 | 1 |
| IPR004092 | Mbt repeat | PF02820 | MBT | 213 | 6 |
| IPR004153 | CXCXC repeat | PF03128 | CXCXC | 59 | 5 |
| IPR004155 | PBS lyase HEAT-like repeat | PF03130 | HEAT_PBS | 1283 | 1 |
| IPR005003 | Phage tail fiber repeat | PF03335 | Phage_fiber | 123 | 1 |
| IPR005100 | Supt5 repeat | PF03439 | Supt5 | 97 | 1 |
| IPR006530 | YD repeat | PF05593 | RHS_repeat | 1450 | 1 |
| IPR006597 | Sel1-like | PF08238 | Sel1 | 3244 | 10 |
| IPR006603 | Cystinosin/ERS1p repeat | PF04193 | PQ-loop | 648 | 6 |
| IPR006624 | Beta-propeller repeat TECPR | PF06462 | Hyd_WA | 108 | 2 |
| IPR006652 | Kelch repeat type 1 | PF01344 | Kelch_1 | 3936 | 72 |
| IPR006691 | DNA gyrase/topoisomerase IV, subunit A, | PF03989 | DNA_gyraseA_C | 2522 | 1 |
| IPR006970 | PT repeat | PF04886 | PT | 291 | 1 |
| IPR007139 | Protein of unknown function DUF349 | PF03993 | DUF349 | 139 | 1 |
| IPR008160 | Collagen triple helix repeat | PF01391 | Collagen | 3340 | 76 |
| IPR008164 | Repeat of unknown function XGLTT | PF01744 | GLTT | 46 | 1 |
| IPR008615 | FNIP | PF05725 | FNIP | 243 | 1 |
| IPR008619 | Filamentous haemagglutinin, bacterial | PF05594 | Fil_haemagg | 581 | 1 |
| IPR008627 | GETHR pentapeptide | PF05671 | GETHR | 64 | 3 |
| IPR009408 | Formin Homology 1 | PF06346 | Drf_FH1 | 23 | 1 |
| IPR010554 | Protein of unknown function DUF1126 | PF06565 | DUF1126 | 129 | 2 |
| IPR010736 | Protein of unknown function DUF1309 | PF07004 | DUF1309 | 114 | 3 |
| IPR010827 | Surface antigen variable number | PF07244 | Surf_Ag_VNR | 1670 | 1 |
| IPR011498 | Kelch repeat type 2 | PF07646 | Kelch_2 | 1098 | 12 |
| IPR011659 | WD40-like Beta Propeller | PF07676 | PD40 | 1997 | 1 |
| IPR013101 | Leucine-rich repeat 2 | PF07723 | LRR_2 | 462 | 1 |
| IPR013105 | Tetratricopeptide TPR2 | PF07719 | TPR_2 | 6902 | 33 |
| IPR013517 | FG-GAP | PF01839 | FG-GAP | 1398 | 8 |
| IPR017868 | Filamin/ABP280 repeat-like | PF00630 | Filamin | 511 | 41 |
| IPR018108 | Mitochondrial substrate/solute carrier | PF00153 | Mito_carr | 5529 | 54 |
| IPR018487 | Hemopexin/matrixin, repeat | PF00045 | Hemopexin | 631 | 7 |
| IPR018502 | Annexin repeat | PF00191 | Annexin | 844 | 6 |
| IPR018942 | Seminal vesicle protein repeat | PF10578 | SVS_QK | 43 | 7 |
| IPR019781 | WD40 repeat, subgroup | PF00400 | WD40 | 19770 | 241 |
